# Vehicle development, pharmacokinetics and toxicity of the anti-invasive agent 4-fluoro-3’,4’,5’-trimethoxychalcone in rodents

**DOI:** 10.1371/journal.pone.0192548

**Published:** 2018-02-22

**Authors:** Liselot M. Mus, Geertrui Denecker, Frank Speleman, Bart I. Roman

**Affiliations:** 1 Center for Medical Genetics, Ghent University Hospital, Ghent, Belgium; 2 Cancer Research Institute Ghent (CRIG), Ghent, Belgium; 3 SynBioC Research Group, Department of Green Chemistry and Technology, Faculty of Bioscience Engineering, Ghent University, Ghent, Belgium; Universidad de Castilla-La Mancha, SPAIN

## Abstract

Effective inhibitors of invasion and metastasis represent a serious unmet clinical need. We have recently identified 4-fluoro-3’,4’,5’-trimethoxychalcone or C16 as a potent anti-invasive molecule. In this paper, we report on the development of an optimized vehicle for oral administration of C16. We also explore its pharmacokinetic and toxicity profile in rodents as a prelude to a broad-scope evaluation as a pharmacological tool in animal models of disease. C16 showed suboptimal pharmacokinetics with limited oral bioavailability and whole blood stability. Rapid metabolism with elimination via glutathione conjugation was observed. An oral dosing routine using medicated gels was developed to overcome bioavailability issues and yielded sustained whole blood levels above the half maximal effective concentration (EC_50_) in a 7-day study. The compound proved well-tolerated in acute and chronic experiments at 300 mg/kg PO dosing. The medicated gel formulation is highly suitable for evaluation of C16 in animal models of disease.

## Introduction

In the malignant progression of a solid tumor the gain of an invasive phenotype is the first and a necessary step in the development of metastases [1,2]. The sequelae of metastasis account for 90% of cancer-related mortality. To date, the biological events underlying invasion and metastasis remain poorly understood [3]. The development of effective anti-invasive/antimetastatic tools and drugs is therefore an unmet clinical need [4].

**Fig 1:**
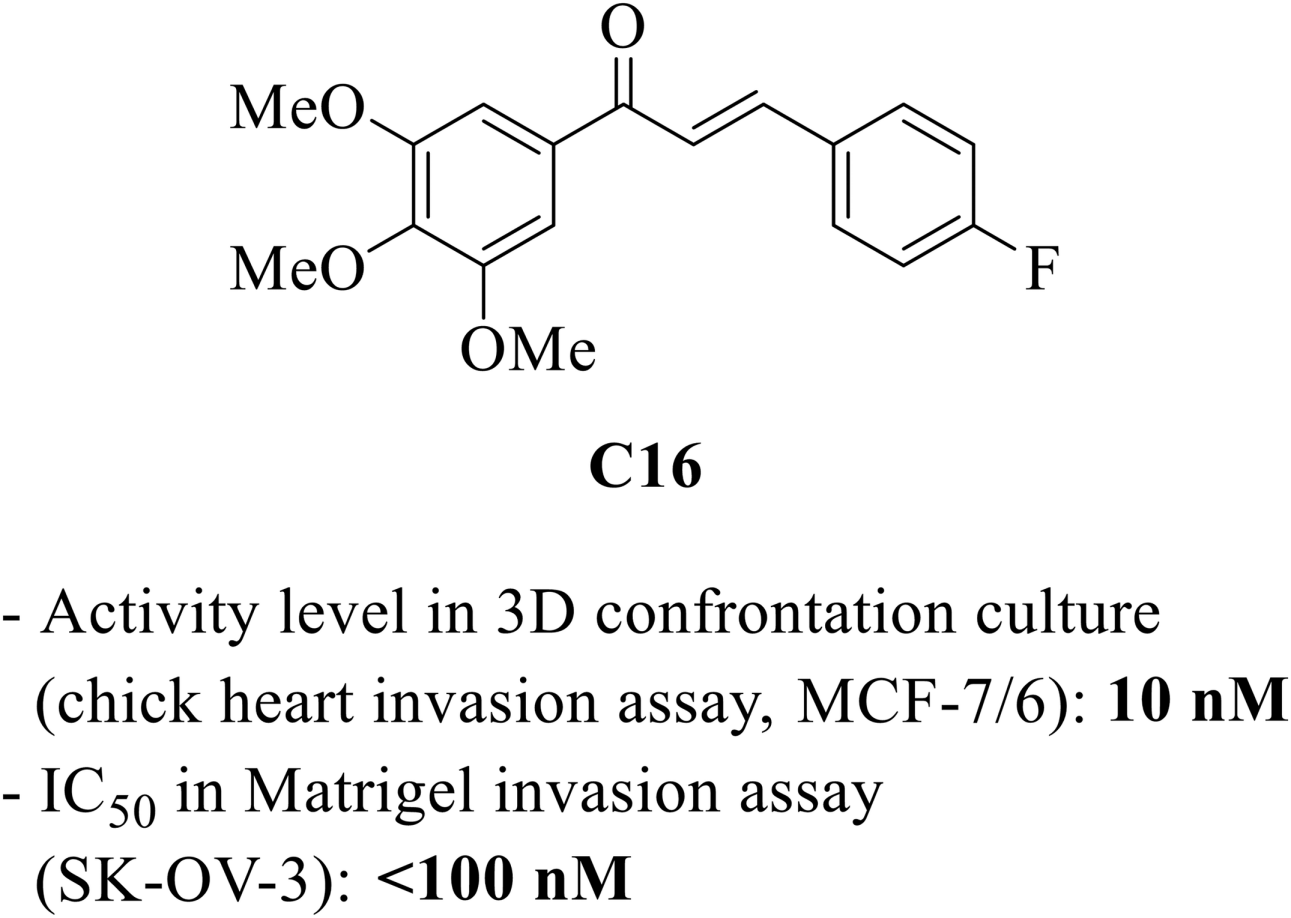
Structure and biological activity of C16.

Our laboratories have been characterizing and validating 4-fluoro-3’,4’,5’-trimethoxychalcone or C16 as a novel invasion inhibitor for the study and potential treatment of metastatic cancer (Fig 1) [5]. C16 has nanomolar anti-invasive potency in the chick heart [6] and Matrigel *in vitro* invasion models against several human cancer cell lines (MCF-7/6 breast cancer, BLM melanoma and SK-OV-3 ovarian carcinoma). The molecule shows a defined structure-activity relationship [7–9] and possesses adequate *in vitro* absorption, distribution, metabolism, and excretion (ADME) properties (*see section ‘Stability and metabolism’ and* [5]). Given its discovery in a phenotypic model, the biological target of C16 is unknown. We have differentiated the molecular mechanism of action of C16 from that of other antimetastatic agents [10], and are currently characterizing its biochemical interactions in detail.

The aim of the present research was to explore the drug metabolism and pharmacokinetics (DMPK) and toxicity profile of C16 in rodents, in preparation of an evaluation of its efficacy as a pharmacological tool in animal models of disease. Here, we have developed appropriate vehicles to accommodate the low bioavailability of C16, including a formulation as a medicated gel suitable for chronic oral dosing. Furthermore, we have analyzed the pharmacokinetic properties and metabolism of C16, as well as its maximum tolerated dose and repeat-dose toxicity profile. Routine pharmacokinetics (PK) and metabolite profiling was conducted in the rat. Additional PK evaluation as well as tolerability and toxicity testing was performed in the mouse, as this species will be used in future proof of efficacy studies.

C16 is a chalcone, a chemical class to which various biological activities have been attributed, albeit often at high concentrations [11,12]. Despite this body of literature, follow-up studies on the DMPK properties of these molecules are rare [13,14], and it thus often remains unclear whether sufficiently high plasma levels can be obtained for a sustained period in order to assess *in vivo* actions. The present study may therefore have relevance to research on other members of the chalcone chemical class.

## Materials and methods

Additional protocols are available in S1 File.

### Statistics

Data was processed using IBM SPSS Statistics 23 or higher. All data were tested for normality (Shapiro-Wilk test) and homogeneity of variances (homoscedasity, Levene's test). If both conditions were met, a one-way ANOVA with post-hoc Tukey hsd was conducted. If the data was not normally distributed, then a non-parametric Kruskal-Wallis H test or Mann-Whitney U test was performed. If the data was not homoscedastic, Welch’s t-test and a post-hoc Gamess-Howell test were conducted.

### Animal welfare

All aspects of this work related to *in vivo* experiments, including housing, experimentation, and animal disposal were performed in general accordance with the *Guide for the Care and Use of Laboratory Animals* (Eighth Edition) [15]. Relevant protocols were approved by the Committee on the Ethics of Animal Experiments of Ghent University (Permit Number: ECD 15/59). The persons who carried out the described experiments received appropriate training in animal care and handling. Animal numbers were kept low due to the exploratory nature of the study. Exact numbers were based on relevant literature work using similar or identical animal studies. Animal health was monitored as indicated in the individual protocols, including during every animal manipulation. Throughout all studies, the following criteria were used to remove an animal from the study and humanely euthanize to prevent undue pain or distress: inability to eat and/or drink, inability to thermoregulate, weight loss (>20%), moribund condition, prolonged bleeding, seizures, paralysis. These endpoints were not reached. No unexpected deaths occurred in any of the described studies. Animals were euthanized by CO_2_, except for those that underwent cardiac exsanguination after Avertin anesthesia. Analgesics or anesthesia were administered as indicated in the individual protocols.

### Chemicals

C16 was prepared as described earlier [5]. A tribromoethanol injectable solution (Avertin) was prepared as described in Section E in S1 File. In-house prepared Milli-Q water was used. Cremophor EL (Sigma, Germany), corn oil (Wako, Japan), dimethyl sulfoxide (DMSO, Sigma-Aldrich, Germany) anhydrous N,N-dimethylacetamide (DMA, Wako, Japan), ethanol (Merck, Germany), Medigel Sucralose (2 oz cups, ClearH2O, USA), 2-methyl-2-butanol (amylene hydrate, Sigma-Aldrich, Germany) 1,2-propanediol (propylene glycol, PG) (Wako, Japan), polyethylene glycol (PEG) 400 (Sigma, U.S.A), PEG 600 (Alfa Aesar, Great Britain or Sigma, USA), sodium heparin (5000 units/mL injectable solution, LEO Pharma, Belgium) Solutol HS-15 (BASF, Germany) 2,2,2-tribromoethanol (Sigma-Aldrich, Germany) were used as obtained from the indicated suppliers.

### Vehicle development

#### Nephelometry

The solubility of C16 was tested at 10 and 30 mg/mL for the indicated vehicles using laser nephelometry in 96-well plates (BMG LabTech NEPHELOstar microplate reader, BMG LabTech, USA). Solutions were prepared using solubilizing techniques including sonication, heating up to 37 °C and vortex. Solubility data was categorized as soluble (result ≤ +15 of blank), slightly soluble (result between +15 and +25 of blank) or not soluble (result > +25 of blank) [16].

#### Preparation of a C16 solution in 20% DMSO / 10% (DMSO / Cremophor EL 1:1) / 70% H_2_O

For a 2 mg/mL final concentration, 240 μL of DMSO was added to 2.4 mg of C16 in a glass vial. The mixture was vortexed resulting in a clear solution. Of this stock solution, 210 μL was mixed with 105 μL of a 1:1 DMSO / Cremophor EL mixture and 735 μL of water, yielding a clear solution.

#### Preparation of a C16 solution in 10% Solutol HS-15 / 90% PEG 600

Solutol HS15 and PEG 600 were warmed to 37 °C. The pre-calculated volume of Solutol HS15 was added to C16. The mixture was vortexed and then kept at 37 °C with sonication until C16 had completely dissolved. PEG 600 was then slowly added and the mixture was vortexed again, whereupon the solution was visually clear. Solutions up to 30 mg/mL were prepared in this way.

#### Stability test of C16 in 10% Solutol HS -15 / 90% PEG 600

Solutions of 30 mg/mL and 10 mg/mL of C16 were prepared and stored at room temperature and 4°C for 24 h. The stability of C16 was evaluated at 0, 1, 2, 4, 8 and 24 h after formulation by high-performance liquid chromatography-mass spectrometry/mass spectrometry (HPLC-MS/MS) (electrospray ionization, ESI+) analysis using oxybutynin as an internal standard (IS). Analyte samples were diluted 500x with acetonitrile (ACN)/H_2_O (20:80). This solution was further diluted 100x with an oxybutynin solution (1 ng/μL in ACN/H_2_O 20:80), giving a final concentration of 0.6 ng/μL and 0.2 ng/μL, respectively, for HPLC-MS/MS analysis (multiple reaction monitoring scan mode, MRM, *see* Section A of S1 File for more details). All analyses were performed in triplicates.

#### Preparation of C16-doped medicated gel

A pre-weighed amount of C16 was dissolved in 1 mL of DMSO and the resulting solution was delivered into a cup of Medigel sucralose (pre-warmed to room temperature) with a syringe through the foil lid. In order to facilitate even distribution of C16 in the gel, the injection was spread over several points. The resulting suspension of C16 in gel was dispersed evenly by vigorous shaking for at least two minutes. Visual control confirmed a uniform and finely dispersed suspension of C16 particles in the gel. The final DMSO concentration in the gel was 1.64%.

#### Tolerability test of vehicles

Vehicles of 10% Solutol HS-15 / 90% PEG 600 and 10% DMA / 90% PEG 600 were evaluated for tolerability by oral gavage at 10 mL/kg using the same protocol as for the single dose *per os* (PO) maximum tolerated dose (MTD) study (*see below*). The tolerability of DMSO-doped medicated gel was evaluated during the repeat-dose toxicity test.

### PK studies

#### Plasma PK study (IV and PO) in male Sprague-Dawley rats

Six male Sprague-Dawley rats (200–350 g) were obtained from Hilltop Labs. Animals were assigned randomly to two groups (IV or PO) of 3 upon arrival. Duration of acclimation was approximately two days. Animals were healthy at the start of the trial.

Animals subjected to IV dosing were fitted with a jugular vein cannula (JVC) under isoflurane anesthesia (induction 4%, maintenance 2.0%, oxygen 1 L/min) and under an external heating source. Post-operative analgesia was provided in the form of 2.5–5 mg/kg of ketoprofen subcutaneously. Benzylpenicillin (60 mg) was given intramuscularly to prevent infection. Sterile 0.9% NaCl was injected subcutaneously under the back skin using a 21G needle during recovery to replace fluid loss. Animals were placed under a heat lamp and monitored until they regained full consciousness. Animals were returned to a sterile cage in the animal care room only after full recovery and when exhibiting normal behavior. Surgically modified animals were housed individually, monitored hourly for the first 4 h and then daily over a 2-day recovery period.

Animals were maintained in a well-controlled temperature (20–24°C) and humidity (30%-70%) environment with 12 h light/dark cycles. Conventional cages with wood chip bedding were used. Animals were identified by a cage label. The study was not blinded. Food was withheld from all animals for a minimum of twelve hours prior to test article administration and returned at approximately 4 h post-dose. Water was supplied ad libitum.

C16 was dosed in cassette with two undisclosed compounds of similar chemical structure. Dosing solutions were prepared in 100% DMSO on the day of dosing. The concentration of C16 was 1 and 5 mg/mL for IV and PO dosing, respectively. C16 was administered at 1 mg/kg IV via JVC or at 10 mg/kg PO.

Sampling took place pre-dose, 5, 15, 30 min, 1, 2, 4, and 8 h post-dose. Blood samples (0.3 mL) were collected via the JVC, placed into chilled tubes containing sodium heparin and kept on ice until centrifugation. Plasma preparation involved centrifugation at a temperature of 2 to 8°C at 3,000 *g* for 5 min. Plasma samples were stored frozen at -70°C until analysis. No necropsy was performed.

#### Whole-blood PK study (IV, IP and PO) in male CD-1 mice

Nine male CD-1 mice (25–35 g) were obtained from Hilltop Labs. Duration of acclimation was at least two days. Animals were randomly assigned to the three dose groups (IV, PO, intraperitoneal (IP), *N* = 3 per group), healthy at the start of the trial, housed one per cage (conventional type with wood chip bedding material), identified by a cage label and maintained in a well-controlled temperature (20–24 C) and humidity (30%-70%) environment with 12 h light/dark cycles. Animals intended for IV dosing were fitted with a jugular vein cannula (JVC, procedure see rat plasma PK study above). The study was not blinded. Food was withheld from the animals for a minimum of 12 h prior to test article administration until 4 h post-dose. Water was offered ad libitum.

C16 was dosed in cassette together with two undisclosed compounds of similar chemical structure. The dose of each test article was 1 mg/kg (IV, JVC, 0.5 mg/mL) or 10 mg/kg (IP and PO gavage, 2 mg/mL). Two respective dosing solutions were prepared (protocol *see above*) containing 0.5 and 2 mg/mL of C16 (and equal concentrations of the other two test articles) in 20% DMSO / 10% (DMSO / Cremophor EL 1:1) / 70% H_2_O. Formulations were prepared one day prior to dosing and stored at 4 °C.

Blood samples were taken pre-dose and 5 (except for PO administration), 15, 30 min, 1, 2, 4, and 8 h after dosing. The samples were collected via the tail vein or by cardiac puncture (8 h time point). Blood (25 *μ*L) was collected and pipetted into a tube with 25 μL heparinized water (1 *μ*L of sodium heparin (1000 unit/ml) + 24 *μ*L water) within 30 seconds of collection. The sample was pipetted up and down five times, immediately frozen on dry ice and stored at -60°C to -80°C until analyzed. No necropsy was performed.

#### Data processing

Pharmacokinetic parameters were estimated by a non-compartmental model using WinNonlin (v5.2.1 or higher) software. The maximum plasma/blood concentrations (*c*_0_) after IV dosing were estimated by extrapolation of the first two time points back to t = 0. The maximum blood concentration (*c*_max_) and the time to reach maximum blood drug concentration (*t*_max_) after PO dosing were derived from the data. The area under the time-concentration curve (AUC) was calculated using the linear trapezoidal rule with calculation to the last quantifiable data point, and with extrapolation to infinity if applicable. Plasma/blood half-life (*t*_1/2_) was calculated from 0.693/slope of the terminal elimination phase. Mean residence time (MRT) was calculated by dividing the area under the moment curve (AUMC) by the AUC. Clearance (CL) was calculated from dose/AUC. Steady-state volume of distribution (*V*_ss_) was calculated from CL*MRT. Bioavailability was determined by dividing the individual dose-normalized PO AUC values by the respective average dose-normalized intravenous (IV) AUC value. Samples that were below the limit of quantification were not used in the calculation of averages, and were treated as zero for pharmacokinetic data analysis.

### Stability and metabolism

For other stability and metabolite experiments, *see* Section C in S1 File.

#### Whole blood stability and metabolite profiling

C16 (1 *μ*M) or vehicle (DMSO) were incubated with fresh rat blood (Sprague Dawley, male) at 37°C at 5 time points in duplicate over a 120-min period. The final DMSO concentration in the incubation was 0.25%. Reactions were terminated following 0, 15, 30, 60 and 120 min by acetonitrile containing internal standard. The sampling plate was centrifuged (3000 rpm, 45 min, 4 °C) and the supernatants from each time point analyzed for parent compound by HPLC-MS/MS. The percentage of parent compound remaining at each time point relative to the 0-min sample was then calculated from HPLC-MS/MS peak area ratios (compound peak area/internal standard peak area).

Following the blood stability assay the time point at which 30–70% of parent had degraded (15-min sample) was analyzed by HPLC-MS over a mass range of 200–1000 Da, using an extended run time. Potential metabolites were identified by searching against expected biotransformations. Unexpected metabolites were also searched for by comparison to a control sample (0-min time point and/or negative control) run under the same analysis conditions. Metabolites had a ratio of at least two compared to a corresponding peak in the control sample.

The metabolite sample was re-injected and data collected for the product ions in the mass window of 50 Da to a point above the metabolite mass, with the precursor ion fixed on the metabolite mass in the time window observed for the metabolite in the original chromatogram. MS/MS spectra of the metabolites were collected and processed into a table representing product ion fragments for the parent and each metabolite.

### Single-dose PO and IP MTD studies

Male ICR mice (20–26 g), were obtained from BioLASCO Taiwan. Animals were kept in conventional cages with wood chip bedding. Space allocation for 3–5 animals was 30 x 19 x 13 cm. All animals were healthy at the start of the trial and maintained in a well-controlled temperature (20–24°C) and humidity (30%-70%) environment with 12 h light/dark cycles. Free access to a standard lab diet [MFG (Oriental Yeast Co., Ltd., Japan)] and autoclaved tap water were granted.

**Fig 2:**
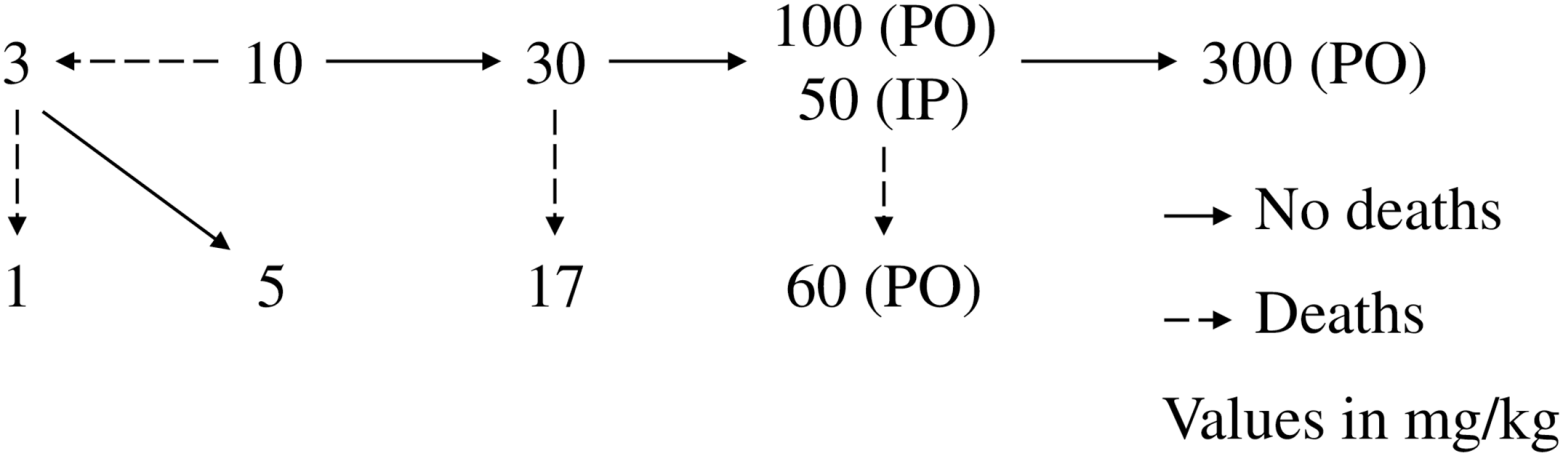
Dose-level decision scheme for the PO and IP MTD studies.

A single PO and IP dose progression test was conducted in which animals were dosed at a minimum of 72- or 24-h intervals, respectively. C16 (vehicle: 10% Solutol HS-15 / 90% PEG 600) was administered by oral gavage at 10 mL/kg to randomly assigned groups of 2 male and 2 female mice, or by IP injection at 5 mL/kg to randomly assigned groups of 5 male mice. Animals received an initial dose of 10 mg/kg. If 50% of the animals survived for 72 h (PO) or 30 min (IP), the dose for the next cohort was increased. If one or more animals died, the dose for the next cohort was decreased. The next dose level was determined by the decision scheme in Fig 2.

The testing stopped when all animals survived at the upper bound, when four (PO) or three (IP) dose levels had been tested or when the upper or lower bound had been reached. At each dose level, animals were observed for the presence of acute toxic symptoms and autonomic effects during the first 60 (PO) or 30 (IP) min, and again at 2, 6 (IP only), 24, 48 (PO only) and 72 h (PO only). Body weights were recorded before dosing and at the 72- (PO) or 24-h (IP) time point. Gross necropsy was performed in all animals without tissue collection.

### 7-Day repeat-dose toxicity study

Fifteen 7-week old male CD-1 mice, weighing 27 to 34 g, were obtained from Janvier, France. Animals were randomly allocated to one of four cohorts (*N* = 4 for the 0 mg/kg, 100 mg/kg and 300 mg/kg C16 cohorts, *N* = 3 for the water control group). Animals were healthy at the start of the trial. Each cohort was housed in one conventional cage with wood chip bedding and kept in a room with a well-controlled temperature (20–24°C) and humidity with 12-h light/dark cycles. Duration of acclimation was five days, during which all animals had ad libitum access to feed and tap water. Feed was obtained from Carfil, Belgium (Complete feed for rats and mice, article n° 10783915). Animals were ear-marked under isoflurane anesthesia (induction 5%, maintenance 2.5%, oxygen 1 L/min) on day -3 of the study. The study was not blinded.

#### Study design

On the morning of day -2 of the study, tap water access was removed from the 0 mg/kg, 100 mg/kg and 300 mg/kg C16 cohorts. These groups were granted access to two non-medicated cups of Medigel sucralose (one in a tailor-made plastic holder with magnetic fixation system to the side of the cage, one in a cardboard holder on top of the bedding material) for an acclimatization period of three days. Note: the magnetic fixation system is preferable, as animals tear and consume the cardboard holders.

Medicated gels were prepared in order to provide animals with a PO C16 dose equaling oral gavage dosing at 0, 100 and 300 mg/kg three times per day (0, 78.43 and 235.29 mg C16 per cup, respectively, assuming a consumption of 7 mL of gel per day and an average animal weight of 30 g). On the morning of day 0 of the study, treatment with two medicated gels was started and maintained up to the morning of day 7 (i.e. a 7-day treatment). Fresh cups were prepared and provided every day. Meanwhile, the water control group was continuously granted unlimited access to tap water. Throughout the study, all animals had ad libitum access to feed, except for the water control group, which was fasted on the evening of day 6, i.e. twelve hours prior to the single PO test article administration. Animal weight, feed and water/gel consumption and the presence of toxic symptoms and autonomic effects were monitored on a daily basis for all cohorts.

On the evening of day 6, blood was collected via the tail vein for the 0, 100 and 300 mg/kg C16 cohorts for determination of the whole blood C16 level. On the morning of day 7, mice of the 0, 100 and 300 mg/kg C16 cohorts were anesthetized by IP injection with 250 mg/kg of Avertin (20 mL/kg, preparation *see* Section E in S1 File). Blood for C16 determination and hematological and biochemical analysis was collected via cardiac exsanguination (600 μL to 1 mL). Detailed analysis protocols are described below. Afterward, animals were sacrificed by cervical dislocation.

On the morning of day 7, the water group received a single PO administration of 300 mg/kg C16 (10 mL/kg) for high-dose PK evaluation, using a 30 mg/mL solution of C16 in 10% Solutol HS-15 / 90% PEG 600 (prepared as described above). To that extent, blood was collected via the tail vein at the 30 and 60 min time point, and via cardiac exsanguination after 180 min (protocol as for other cohorts). Samples for hematological and biochemical analysis were also prepared. Afterward, animals were sacrificed by cervical dislocation.

All animals were necropsied and assessed for gross pathology. Liver, kidneys and spleen weights were recorded, and fragments of these organs were fixed in a 4% formaldehyde solution and processed for microscopic evaluation.

#### Whole blood C16 determination

Blood samples (25 *μ*L) were collected via the tail vein or cardiac puncture (last time point). Blood was pipetted into a tube with 25 *μ*L heparinized water (1 *μ*L of sodium heparin (1000 units/mL) + 24 *μ*L water) within 30 seconds of collection. The sample was pipetted up and down five times, immediately frozen on dry ice, and stored at -60°C to -80°C until analysis.

#### Hematology and biochemical analysis

Cardiac puncture as described above afforded 0.6–1 mL of blood. Approximately 500 *μ*L thereof was collected in a Greiner Bio 0.5 mL ethylenediaminetetraacetic acid (EDTA) Eppendorf tube for hematological parameter determination and kept at room temperature. The remaining volume was collected in a regular Eppendorf tube and allowed to clot completely at room temperature over 45 min. The tubes were centrifuged at 1,900 x *g* (3000 rpm) and 4 °C for 10 min using a swinging bucket rotor. The upper serum phase was transferred to a new tube with conical bottom. Care was taken not to disturb the intermediate buffy coat layer. The serum samples were centrifuged at 16,000 x *g* and 4 °C for 10 min in a fixed-angle rotor. The cleared supernatant was transferred to a new tube without disturbing the pellet, affording 200–500 *μ*L of serum. Samples were diluted to 500 *μ*L with normal saline, flash frozen in liquid N_2_ and stored at -80 °C prior to analysis. Hematological analysis was conducted on an XE-5000 Automated Hematology System (Sysmex, USA), biochemical analysis on a cobas 8000 series system (Hitachi/Roche, USA).

## Results

### Vehicle development

#### Vehicles for single-dose studies

C16 has low aqueous solubility, which limits the choice of preclinical formulations for *in vivo* studies. In this work, thirteen vehicles were evaluated for their applicability in single-dose studies (Table 1). For commonly used systems (entries a-e) visual determination of solubility was conducted, while for the more complex systems solubility was assessed by laser nephelometry.

**Table 1:** Solubility of C16 in vehicles suitable for *in vivo* evaluation.

		Concentration (mg/mL)	Result
a	DMSO	10	**Soluble** ^a^
b	10% DMSO in normal saline	1	Insoluble ^a^
c	50% DMSO in normal saline	1	Insoluble ^a^
d	10% (DMSO / Cremophor EL 1:1) / 90% Milli-Q water	2	Insoluble ^a^
e	20% DMSO / 10% (DMSO / Cremophor EL 1:1) / 70% Milli-Q water	2	**Soluble** ^a^
f	10% Cremophor EL / 10% EtOH	10	Insoluble ^b^
g		30	Insoluble ^b^
h	10% DMA / 10% EtOH / 20% PG	10	Insoluble ^b^
i		30	Insoluble ^b^
j	10%DMA / 20%PG / 40% PEG 400	10	Insoluble ^b^
k		30	Insoluble ^b^
l	40% PEG 400 / 10% EtOH	10	Insoluble ^b^
m		30	Insoluble ^b^
n	20% PEG 400 / 10% Cremophor EL	10	Insoluble ^b^
o		30	Insoluble ^b^
p	10% Solutol HS-15 / 90% PEG 600	10	**Soluble** ^b^
q		30	**Soluble** ^b^
r	100% Corn oil	10	Insoluble ^b^
s		30	Insoluble ^b^
t	10% Dimethylacetamide (DMA) / 90% PEG 600	10	**Soluble** ^b^
u		30	**Soluble** ^b^

^*a*^ Visual determination.

^*b*^ Laser nephelometry.

**Fig 3:**
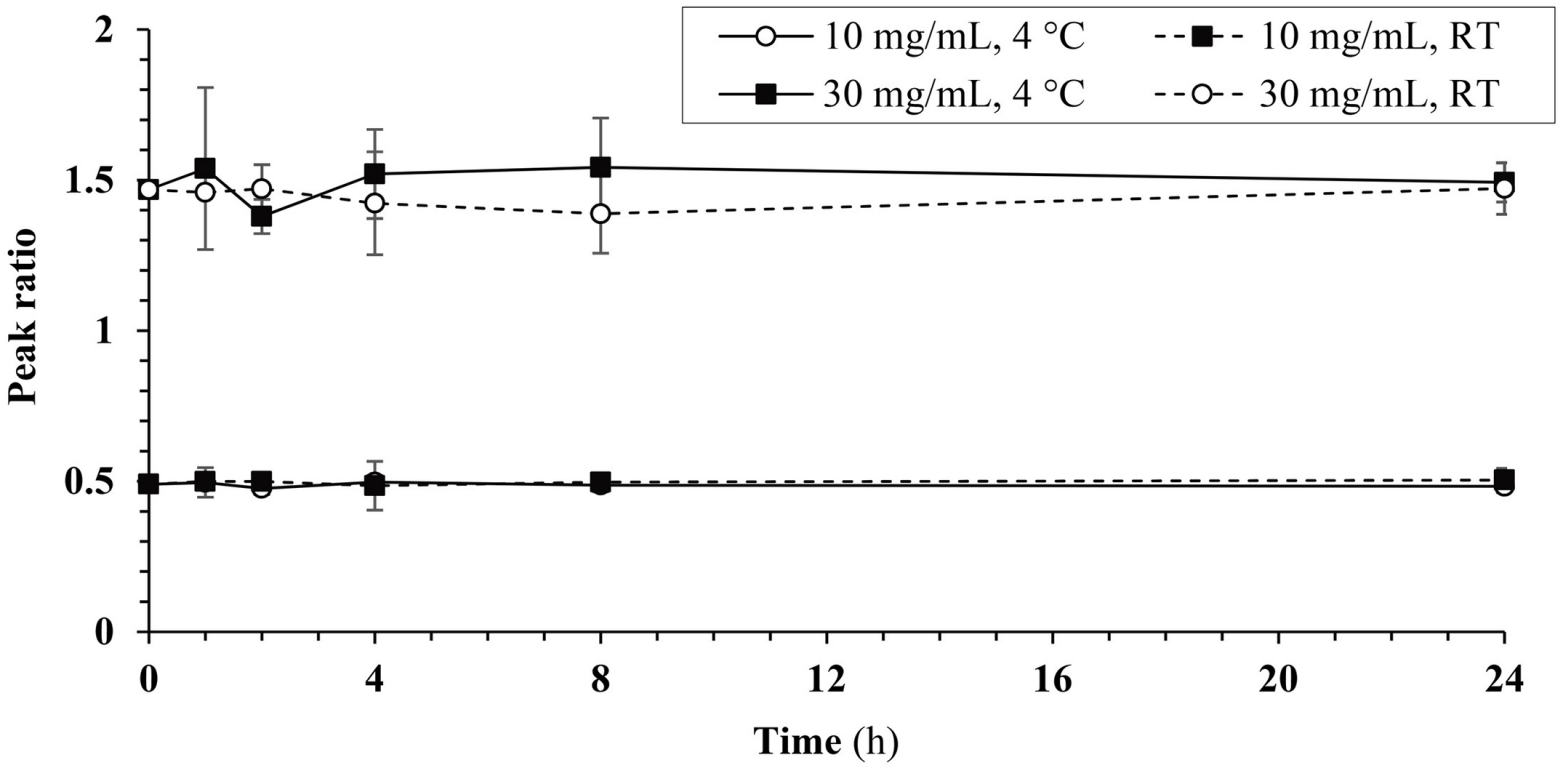
Stability of C16 in 10% Solutol HS-15 / 90% PEG 600 at 4 °C and room temperature (RT). Peak ratios of C16 versus IS are shown (ANOVA/Tukey HSD or Welch/Gamess-Howell).

As expected, C16 proved highly soluble in DMSO (entry a). A more preferable formulation with 25% DMSO in Cremophor and water (entry e) also proved suitable at low concentrations (up to 2 mg/mL). These vehicles are appropriate for single-dose studies with low amounts of C16, e.g. in PK studies. Dimethylacetamide / 90% PEG 600 and Solutol HS-15 / PEG 600 gave clear solutions at 10 and 30 mg/mL (entries p,q and t,u). These potential high-dose vehicles were further evaluated for tolerability and stability. Solutions of 10 and 30 mg/mL C16 in 10% Solutol HS-15 / 90% PEG 600 proved stable at both room temperature (20–25 °C) and 4°C over 24 h (Fig 3, ANOVA/Tukey HSD or Welch/Gamess-Howell, Table A in S1 File). No adverse signs were elicited by both vehicles on PO administration (*see section* ‘*Single dose PO and IP MTD studies’*). IP dosing of the more common of these two vehicles, Solutol HS-15 / PEG 600, at 5 mL/kg induced mild adverse effects (decreased exploratory behavior, decreased muscle tone) within 30 min after administration (*see section* ‘*Single dose PO and IP MTD studies’*, 10% DMA / 90% PEG 600 not tested). 10% Solutol HS-15 / 90% PEG 600 thus is a valid vehicle for high-dose experiments, e.g. MTD studies.

#### Vehicle for repeat-dose studies

**Fig 4:**
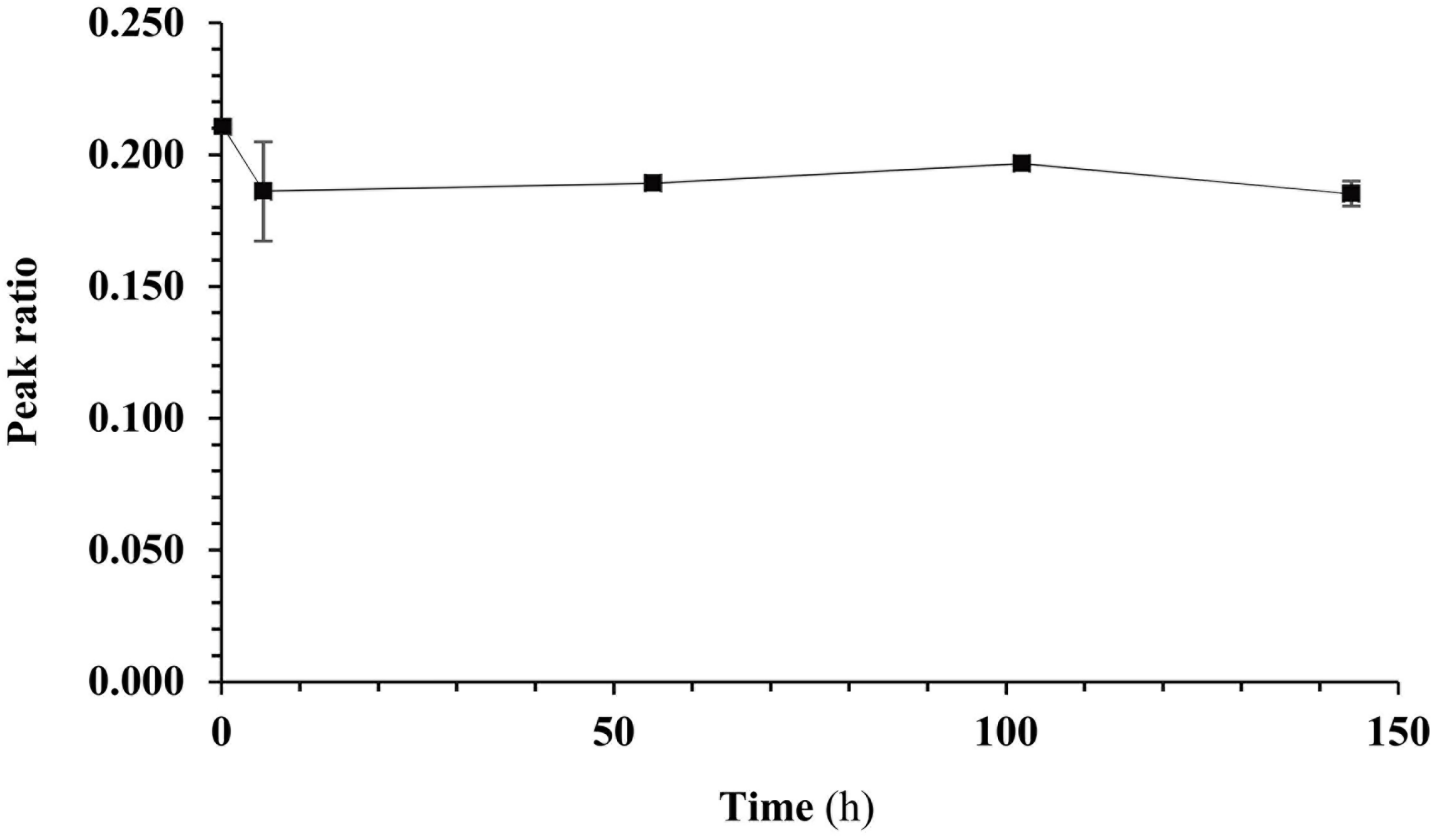
Stability of a dispersion of C16 in Medigel Sucralose (235.29 mg/cup) containing 1.64% DMSO at room temperature. Peak ratios of C16 and IS are displayed (average ± SD of 3 wavelengths: 220 nm, 254 nm and 280 nm).

For repeated oral dosing over an extended period of time, a formulation of C16 in medicated gel (Medigel Sucralose, ClearH2O) was developed as an alternative to repeated oral gavage. Medigel Sucralose is a non-wetting sucralose-flavored, low calorie water gel with a sweet taste. The chosen formulation as a medicated gel is a stable suspension of C16 particles in water. The stability of this dispersion at room temperature was confirmed over 144 h (Fig 4 and Tables C-D in S1 File), and the compound proved uniformly distributed (*see* Table E in S1 File). The tolerability of Medigel Sucralose had been explored by others [17]. Observations on the tolerability of DMSO in the gel (this co-solvent is used as a solubilizing agent during preparation) are mentioned in the discussion of the repeat-dose toxicity test.

### PK parameters of C16 in the rat and mouse

**Fig 5:**
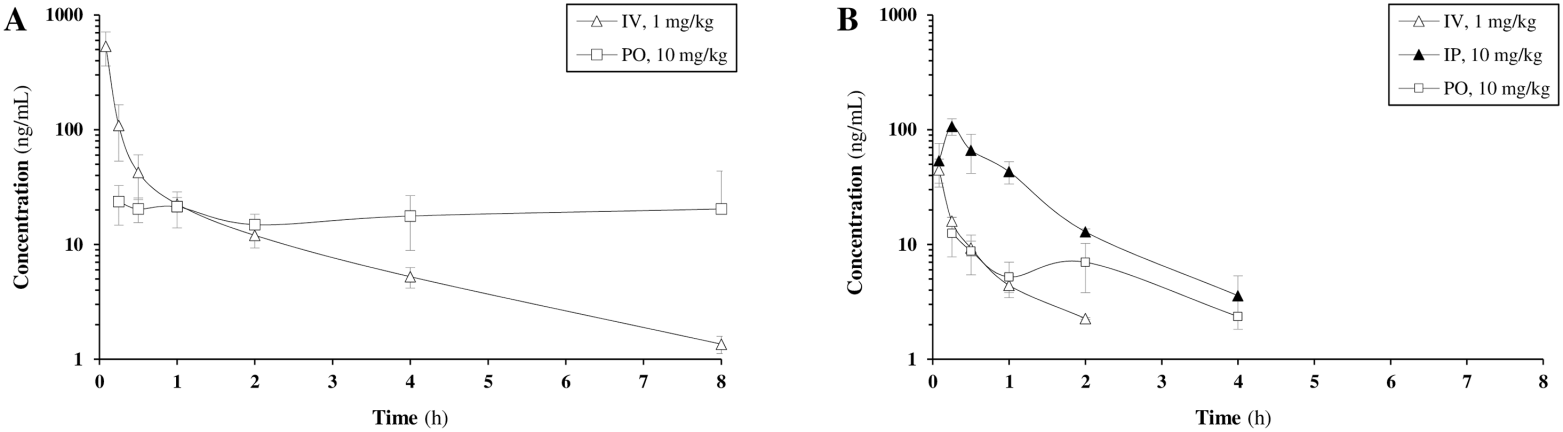
C16 Pharmacokinetics: Concentration-time curves. A: rat, plasma; B: mouse, whole blood.

C16 plasma pharmacokinetics were determined in male Sprague-Dawley rats after intravenous and oral administration at 1 and 10 mg/kg, respectively (Table 2, Fig 5A, Tables F-G and Figs B-C in S1 File). In this exploratory experiment, C16 and two other (undisclosed) compounds were dosed in cassette in 100% DMSO. No adverse effects were observed after the intravenous and oral administration of the materials. Following intravenous dosing of C16 at 1 mg/kg, a *c*_0_ of 1257 ng/mL was reached with an average half-life of 1.93 h. The average clearance was 4.95 L/h/kg, the volume of distribution 4.77 L/kg. After oral dosing at 10 mg/kg, a *c*_max_ of 32.2 ng/mL and oral bioavailability of 6.91% was found. The half-life value of 3.67 h was longer than that observed after IV administration.

**Table 2:** Average PK parameters of C16 in the mouse and the rat.

	Plasma, male Sprague-Dawley rat		Whole blood, male CD-1 mouse		
	IV	PO	IV	IP	PO
Dose (mg/kg)	1 ^a^	10 ^a^	1 ^b^	10 ^b^	10 ^b^
*c*_0_ (ng/mL) ^c^	1257		76.0		
*c*_max_ (ng/mL) ^c^		32.2		107	12.5
*t*_max_ (h) ^c^	0	2.83	0	0.25	0.25
*t*_1/2_ (h)	1.93	3.67 ^f^	0.746	0.800	ND
MRT_last_ (h)	0.76	3.67	0.433	0.800	1.38
CL (L/h/kg)	4.95		45.0	0.952	
*V*_ss_ (L/kg)	4.77		32.6		
AUC_last_ (h∙ng/mL)	211	146	20.0	109	20.9
AUC_∞_ (h∙ng/mL)	215	161 ^f^	22.4	114	ND
**Dose-normalized values** ^d^					
AUC_last_ (h∙kg ng/mL/mg)	211	14.6		10.9	2.09
AUC_∞_ (h∙kg ng/mL/mg)	215	16.1 ^f^		11.4	ND
Bioavailability (%) ^e^		6.91		54.7	10.5

*c*_0_: Maximum plasma concentration extrapolated to *t* = 0; *c*_max_: Maximum plasma concentration; *t*_max_: Time of maximum plasma concentration; *t*_1/2_: half-life; MRT_last_: Mean Residence Time, calculated to the last observable time point; CL: Clearance; *V*_ss_: Steady state volume of distribution; AUC_last_: Area under the curve, calculated to the last observable time point; AUC_∞_: Area under the curve, extrapolated to infinity; ND: not determined.

^*a*^ Vehicle: DMSO.

^*b*^ Vehicle: 20% DMSO/10% (DMSO/Cremophor EL 1:1)/70% H_2_O.

^*c*^ Extrapolated to *t* = 0.

^*d*^ Dose normalized by dividing the parameter by the nominal dose in mg/kg.

^*e*^ Bioavailability determined by dividing the individual dose normalized oral AUC by the individual dose normalized intravenous AUC value;

^*f*^ Calculated for two out of three animals.

Next, the whole-blood pharmacokinetics of C16 in male CD-1 mice were evaluated after intravenous (1 mg/kg), intraperitoneal (IP, 10 mg/kg) or oral (PO, 10 mg/kg) administration in cassette dosing with two other (undisclosed) compounds (Table 2, Fig 5B, Tables H-J and Figs D-F in S1 File). In this experiment, 20% DMSO/10% (DMSO/Cremophor EL 1:1)/70% H_2_O was used as a more preferable vehicle with respect to pure DMSO. No adverse reactions were observed. Following intravenous (IV) dosing at 1 mg/kg, C16 had a half-life of 0.746 h, a clearance rate of 45.0 L/h/kg, and a volume of distribution of 32.6 L/kg. Following IP and PO dosing at 10 mg/kg, C16 reached a maximum blood concentration of 107 and 12.5 ng/mL, respectively, both at 15 min post dosing. The average IP and PO bioavailability was 54.7% and 10.5%, respectively.

### Stability and metabolism

Interpretation of the above PK data required the generation of additional *in vitro* stability and metabolism data (Table 3), and revision of known data [5]. As illustrated above, solubility and lipophilicity are acceptable for exploratory work *in vivo* when using suitable vehicles. C16 has a high Caco-2 classification, with a discrepancy in recovery for the A→B and B→A-directions.

**Table 3:** *In vitro* physicochemical, stability and metabolism data.

Parameter		Unit	Value
**Aqueous solubility** ^a^	PBS, pH 7.4	*μ*M	7.9
	simulated intestinal fluid	*μ*M	2.4
	simulated gastric fluid	*μ*M	9.1
**LogD** ^b^^,^^f^			3.85
**Protein binding and recovery** ^b^^,^^g^		%	99–90
**Permeability and recovery** ^c^^,^^h^	A-B	10^−6^ cm/s—%	10.4–17
	B-A	10^−6^ cm/s—%	4.9–66
**Half-life**	PBS, pH 7.4 ^d^	min	>240
	simulated gastric fluid ^d^	min	>60
	simulated intestinal fluid ^d^	min	>240
	blood (rat, Sprague-Dawley) ^d^	min	6
	plasma (rat, Sprague-Dawley) ^d^	min	>120
	liver microsomes (rat, Sprague-Dawley) ^e^	min	20
**Intr. clearance**	liver microsomes (rat, Sprague-Dawley) ^e^	μL/min/mg	339
**CYP inhibition**	1A2 (CEC substrate) ^c^	% inhibition of control	36
	2D6 (MFC substrate) ^c^	% inhibition of control	-19
	3A4 (BFC substrate) ^c^	% inhibition of control	27
**Blood partitioning (rat, Sprague-Dawley)** ^d^			9.2

CYP: cytochrome P450.

Test concentration:

^*a*^ 200 μM;

^*b*^ 100 μM;

^*c*^ 10 μM;

^*d*^ 1 μM;

^*e*^ 0.1 μM;

^*f*^ Partition coefficient n-octanol/PBS, pH 7.4;

^*g*^ Human plasma;

^*h*^ Caco-2, pH 7.4/7.4.

The compound is stable in simulated intestinal and gastric fluid and in PBS. A much higher intrinsic clearance was obtained for rat liver microsomes in comparison with earlier results for humans [5]. C16 does not significantly inhibit three major drug-drug interaction (DDI)-inducing human cytochromes P450 (CYPs, 1A2, 2D6 and 3A4). Despite the high protein binding of 99%, C16 has a high blood-to-plasma ratio of 9.2. A remarkable difference in half-life in rat plasma and whole blood was obtained. In the whole blood stability assay, comparison of the 15-min sample against the 0-min control revealed one major metabolite (Table 4, Tables M-P in S1 File). HPLC-MS/MS profiling showed that this compound had the mass of a C16-glutathione conjugate. Remarkably, also the *Z*-isomer of C16 was detected in this experiment. Fast partial isomerization apparently occurred under the conditions of this experiment, as the configuration of C16 was initially purely *E* (confirmed by nuclear magnetic resonance (NMR) analysis). This isomerization was also observed after prolonged dissolution in Medigel Sucralose (*see below* and Table C in S1 File). A similar isomerization was also made by Gutteridge et al. for two other chalcones [18]. Both the *E*- and *Z*-isomer of C16 showed a comparable short half-life in rat whole blood. The extent to which this isomerization occurs *in vivo* is unknown, yet it does not cause tolerability issues (*see below*).

**Table 4:** Parent/metabolite areas and percentage in 0 min control and 15 min samples.

	Area in sample (%)	
	0 min control	15 min sample
C16	62.4	52.2
*Z*-isomer	36.5	10.7
Glutathione conjugate	1.06	37.1

### Single-dose PO and IP MTD study

The MTD of C16 in mice, using 10% Solutol HS-15 / 90% PEG 600 as a high-dose vehicle, was determined for both PO and IP administration. PO administration (oral gavage) was evaluated in male and female ICR mice (*N* = 2 per group) using a starting dose of 10 mg/kg. The compound was tolerated at 10–300 mg/kg though mild adverse effects such as vocalization and increase in sensitivity to touch were observed at 100 and 300 mg/kg (Table 5, *see also* Section D in S1 File). All animals survived during the 72-h observation period and no gross lesions were discovered after necropsy. Body weight gain was not affected between cohorts of the same sex (Gamess-Howell).

**Table 5:** MTD study in mice, PO administration: Observations.

Treatment	Vehicle ^a^		C16							
	10 mL/kg		10 mg/kg		30 mg/kg		100 mg/kg		300 mg/kg	
Sex	M	F	M	F	M	F	M	F	M	F
Behavioral ^b^^,^^c^	none	none	none	none	none	none	Yes: A,C	Yes: B,C	Yes: A	Yes: A
Neurologic ^c^	none	none	none	none	none	none	none	none	none	none
Autonomic ^c^	none	none	none	none	none	none	none	none	none	none
Deaths	0/2	0/2	0/2	0/2	0/2	0/2	0/2	0/2	0/2	0/2
Necropsy: gross findings ^d^	none	none	none	none	none	none	none	none	none	none
Weigt increase (%) ^e^	21±0	4±6	25±3	2±3	18±3	6±3	25±1	8±1	17±0	7±3

^*a*^ Vehicle: 10% Solutol HS-15 / 90% PEG 600.

^*b*^ Behavioral changes: A: Irritability: vocalization, 1/2; B: Irritability: vocalization, 2/2; C: Slightly increased touch, 1/2.

^*c*^ Observations made over the first 60 min.

^*d*^ Necropsy 72 h post dosing.

^*e*^ Over 72 h (average±SD)

C16 was also administered intraperitoneally (IP) to a group of 5 male ICR mice. The vehicle 10% Solutol HS-15 / 90% PEG 600 at 5 mL/kg induced adverse effects such as decreased exploratory behavior and decreased muscle tone within 30 min after administration (Table 6 and Section D in S1 File). Additional effects such as decreased sensitivity to touch, decreased exploratory behavior, decreased spontaneous activity, decreased muscle tone, deep respiration, decreased palpebral size and hunch back were elicited by C16 at the three doses levels (10, 30 and 50 mg/kg), with more severe effects observed at higher doses. The effects were reversible, and all animals survived without significant changes in food consumption across groups (Kruskal-Wallis) and with moderate weight decrease during the 24-hour experimental period. Only between the 10 and 50 mg/kg cohorts, a significant difference in average weight decrease was noted (*p* = 0.037, Kruskal-Wallis). In addition, no significant changes were observed during gross necropsy.

**Table 6:** MTD study in mice, IP administration: Observations.

Treatment	Vehicle ^a^	C16		
	5 mL/kg	10 mg/kg	30 mg/kg	50 mg/kg
**Behavioral** ^b^^,^^c^				
Irritability: vocalization	0/5	0/5	1/5, ±	1/5, ±
Decreased touch response	1/5, ±	3/5, ±	5/5, ±	5/5, ±
Decreased exploration	3/5, ±	2/5, ±	3/5, ±	3/5, ±
Pinna	0/5	0/5	2/5, ±	2/5, ±
**Neurologic** ^b^^,^^c^				
Tremor: twitches	1/5	2/5	2/5	3/5
Decreased spontaneous activity	1/5, ±	3/5, ±	4/5, ±	5/5, ±
Ataxia	2/5, ±	2/5, ±	3/5, ±	2/5, ±
Low limb post	0/5	0/5	1/5, ±	2/5, ±
Abdominal tone	4/5, ±; 1/5, +	1/5, ±; 4/5, +	2/5, ±; 3/5, +	3/5, ±; 2/5, +
Limb tone	2/5, ±; 3/5, +	1/5, ±; 4/5, +	2/5, ±; 3/5, +	5/5, +
Grip strength	5/5, ±	4/5, ±	3/5, ±; 1/5, +	5/5, ±
**Autonomic** ^b^^,^^c^				
Respiration: depth	3/5, ±	3/5, ±	5/5, ±	3/5, ±
Low body temperature	0/5	0/5	0/5	5/5, ±
Decreased palpebral size	0/5	2/5, ±	4/5, ±; 1/5, +	5/5, ±
Hunch back	0/5	3/5	5/5	5/5
**Deaths**	0/5	0/5	0/5	0/5
**Necropsy: gross findings** ^d^	none	none	none	none
**Weigt decrease** (%) ^e^	5.1	2.4	4.8	8.7

±: slight to moderate; +: severe;

^*a*^ Vehicle: 10% Solutol HS-15 / 90% PEG 600.

^*b*^ For all observed criteria, *see* Table U in S1 File.

^*c*^ Observations made over the first 30 min.

^*d*^ Necropsy 24 h post dosing.

^*e*^ Over 24 h (average±SD).

### Repeat-dose toxicity study

Finally, an assessment was conducted of the suitability of the medicated gel as a vehicle for oral delivery, and of the toxicity of C16 on continuous dosing via this route of administration. Animals were given the equivalent of *ter in die* (TID, three times per day) dosing of 0, 100 and 300 mg/kg of C16 in Medigel Sucralose (1.64% DMSO) over a 7-day period. The 300 mg/kg value was chosen as it was the highest evaluated dose in the single-dose MTD study. As a control, a fourth cohort received regular drinking water during the same period. Overall, the Medigel vehicle and compound were well-tolerated (raw data is available in Section E in S1 File). All animals survived during the 7-day observation period.

**Fig 6:**
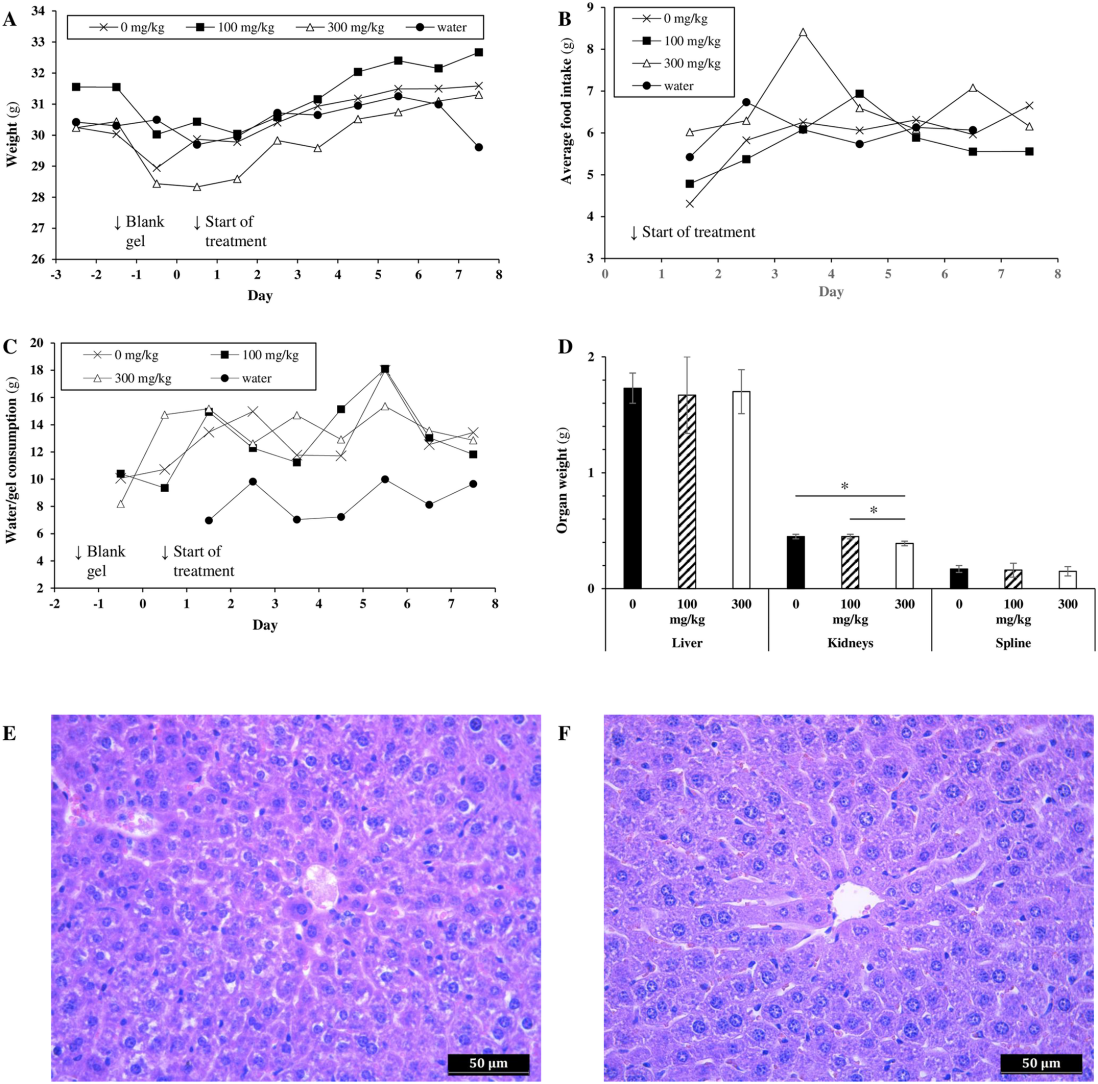
Selection of parameters recorded during the repeat-dose toxicity study. A. Animal weight (ANOVA, Kruskal-Wallis, error bars were omitted for clarity, see Table V in S1 File). B. Feed consumption (Tukey HSD). C. Daily gel or water consumption (Tukey HSD). D. Average organ weights ±SD (ANOVA with post-hoc Tukey HSD; *p = 0.001). E. Hepatocyte toxicity, 0 mg/kg cohort animal. F. Normal hepatocytes, 300 mg/kg cohort animal.

As seen in the single-dose MTD study, mild behavioral changes were observed in the C16-treated cohorts. From day 2 on, animals exhibited hyperactivity and increased anxiety on opening of the cages. This behavior was absent in the 0 mg/kg and the water groups. The effects were mild, ranging from slightly noticeable in the 100 mg/kg cohort to noticeable in the 300 mg/kg cohort. The magnitude of these effects did not increase in time. No significant difference in average weight between the four cohorts was noted during the study (Fig 6A, Tukey HSD or Kruskal-Wallis). A slight but non-significant decrease in weight was observed during the acclimatization period for all three Medigel cohorts with respect to the water cohort, indicating slight neophobia towards the non-medicated gel. Animals recovered fast from day 0–1 on, despite the start of the treatment (no neophobia towards C16 or DMSO). Average food intake per animal was around 6 g/day with a slight upward trend over the test period in all cohorts (Fig 6B). Throughout the study, no significant differences in average food consumption were observed between study groups (Tukey HSD) [19]. Decrease of gel per day was between 8 and 16 g/animal, and did not differ significantly between the gel cohorts (Fig 6C, Tukey HSD). An identical, slowly increasing trend in consumption was observed. The actual gel consumption lies a fraction lower than the reported amounts because of loss of gel due to sanitation of the cups pre-weighing. We estimate that the actual consumption will be close to that of the water cohort, which is commensurate with the manufacturer’s findings [20].

No gross lesions were discovered on detailed necropsy. Liver and spleen weights were identical across the Medigel-treated cohorts, while a slight but significant decrease in kidney weight was noted for the 300 mg/kg cohort with respect to the 100 mg/kg and to the 0 mg/kg cohort (Fig 6D, ANOVA with post-hoc Tukey HSD, *p* = 0.001). No comparison was made with the water cohort, since these animals were fasted overnight prior to necropsy. Histology of the kidney and spleen revealed no abnormalities. A slight hepatocyte toxicity (centrilobular) was noted in some animals of the Medigel-treated groups, but not in the water cohort (Fig 6E & 6F).

Clinical chemistry and hematology parameters were determined in all cohorts at the end of the study (Table 7). All measurements fell within or were close to normal ranges (*see* Table Z in S1 File). A (marginally) statistically significant difference between cohorts was only noted for the glucose measurements of the treatment groups versus the blank. This result was expected, since only the blank animals were fasted overnight prior to analysis.

**Table 7:** Clinical chemistry and hematology results after 7-day continuous PO dosing via medicated gels versus the water control group(average±SD).

Parameter	Cohort			
	0 mg/kg	100 mg/kg	300 mg/kg	water
**Clinical chemistry**				
ALP (IU/L)	92±12	118±33	94±18	93±14
ALT (IU/L)	43±19	70±40	43±9	78±36
AST (IU/L)	188±84	345±365	221±65	466±387
CHO (mg/dL)	91±17	96±21	73±18	91±8
GLUC (mg/dL) *^,^^a^	206±11 ^a^	218±30 *	206±24 ^a^	136±57
TP (g/L)	37.8±1.0	42.0±4.5	31.6±21.3	45.7±3.6
TRIGL (mg/dL)	207±79	110±45	123±55	108±21
**Hematology**				
WBC (10^3^/*μ*L)	3.02±0.77	3.05±0.08	2.97±0.94	1.62
RBC (10^6^/*μ*L)	7.02±1.40	8.32±0.69	6.32±1.92	8.09
HGB (g/dL)	11.3±2.0	12.2±0.2	10.1±2.9	13
HCT (%)	37.7±7.1	44.5±5.2	34.0±7.78	40.9
MCV (fL)	53.8±0.6	53.4±1.8	54.5±4.24	50.6
MCH (pg)	16.2±0.4	14.7±1.5	16.0±0.2	16.1
PLT (10^3^/*μ*L)	258±41	281±33	2665±12	338
NEUT (%)	28.6±4.7	16.9±4.9	39.9	43.2
LYMP (%)	65.4±9.9	79.2±3.4	60.1	56.8
MONO (%)	1.4±1.3	2.5±0.6	2.6±3.7	0
EO (%)	0.4±0.5	0±0	0±0	0
BASO (%)	0.08±0.2	0.2±0.2	0±0	0
NRBC (10^3^/*μ*L)	0±0	0.75±1.06	0.35±0.49	1.2
IG (10^3^/*μ*L)	0±0	0.04±0.06	0	0

Abbreviations: WBC: white blood cells; RBC: red blood cells; HGB: hemoglobin; HCT: hematocrit; MCV: Mean corpuscular volume; MCH: Mean corpuscular hemoglobin; PLT: platelets; PCT: platelet hematorcrit; ALP: alkaline phosphatase; ALT: alanine transaminase; AST: aspartate transaminase; CHO: cholesterol;; GLUC: glucose; IG: immature granulocytes; NRBC: nucleated red blood cells; TP: total protein; TRIGL: triglicerides;

* Significant difference in glucose level between 100 mg/kg and water cohort (*p* = 0.028);

^*a*^ Marginally significant difference between 0 mg/kg and blank cohort (*p* = 0.062), and between 300 mg/kg and blank cohort (*p* = 0.063).

**Fig 7:**
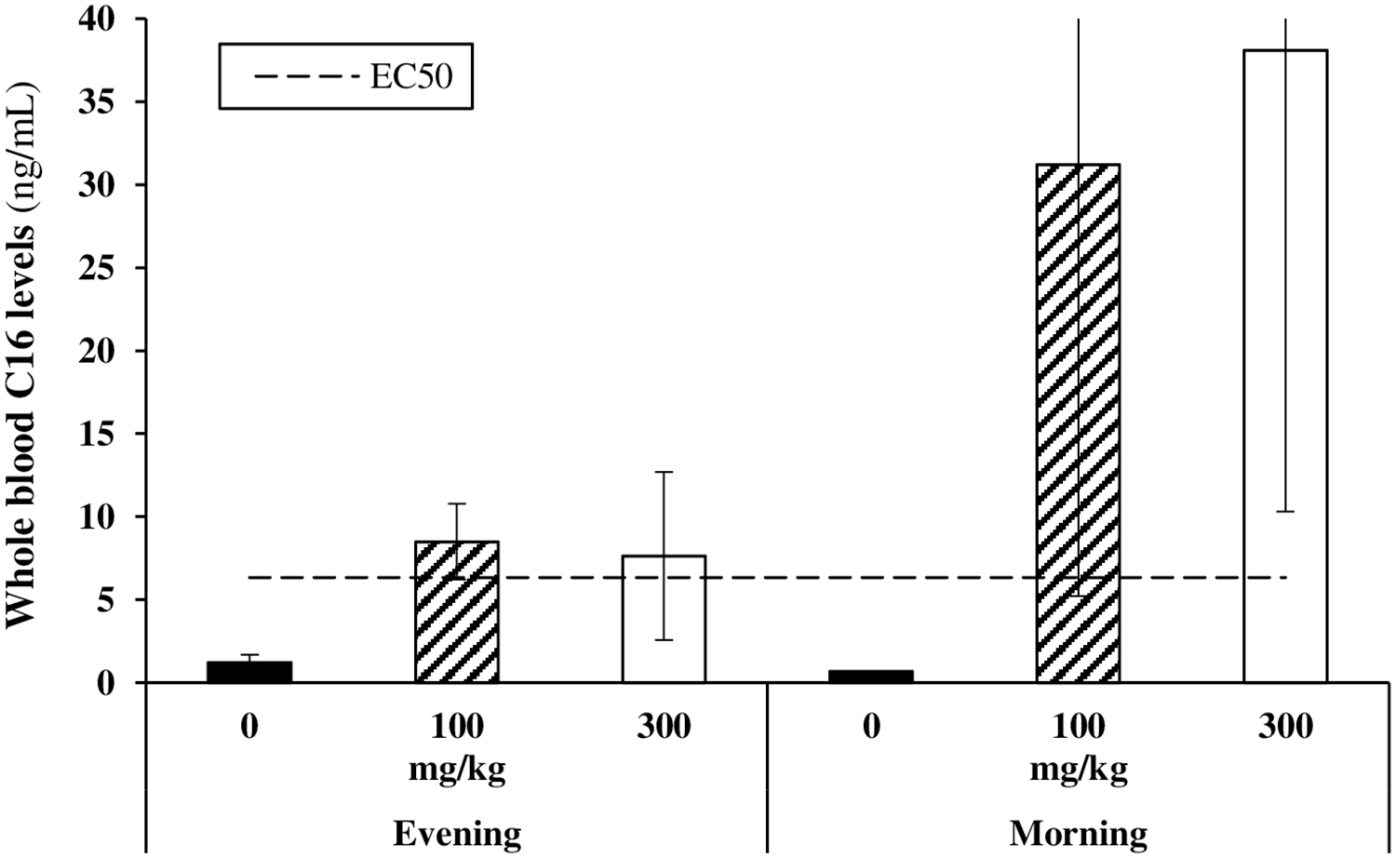
Whole blood levels of C16 after 7-day continuous PO dosing via medicated gels and comparison with projected C16 EC_50_ value (10 nM or 6.32 ng/mL). *N* = 4, except for the 300 mg/kg evening condition (*N* = 3). Respectively 2 and 3 animals had C16 levels below the limit of quantification in the 0 mg/kg evening and morning conditions. Statistics: ANOVA or Mann-Whitney U test.

On the last evening (day 6) and last morning (day 7) of the study, blood samples were taken from the Medigel-treated cohorts to determine the systemic availability of C16 via this route of administration. At both time points, whole blood C16 concentrations were above its projected EC_50_ (Fig 7). No difference was found in C16 levels between the 100 mg/kg and 300 mg/kg cohort, in both the evening (ANOVA) and morning (Mann-Whitney U test) samples. As expected from their dietary pattern, average C16 levels were higher in the morning than in the evening. Nevertheless, this difference was not statistically significant for the 100 mg/kg cohort (*p* = 0.200, Mann-Whitney U test) and only borderline significant for the 300 mg/kg group (*p* = 0.057, Mann-Whitney U test).

**Fig 8:**
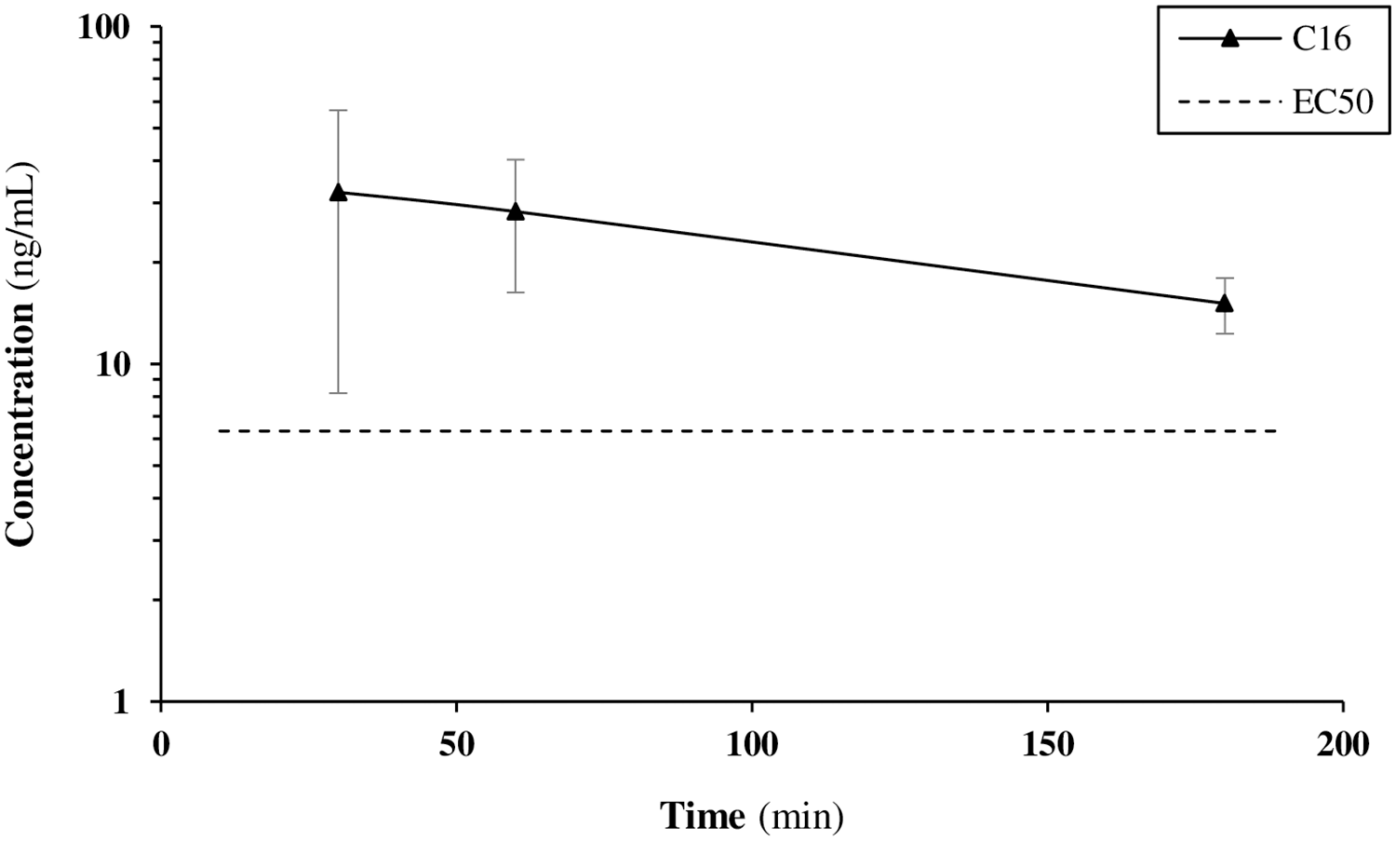
Concentration-time curve for C16 on dosing via oral gavage at 300 mg/kg in the mouse.

A final aspect of this 7-day study was a comparison between attainable plasma levels of C16 on administration *via* gel and oral gavage. On the final morning of the study, animals in the water cohort received a single PO administration of 300 mg/kg of C16 in 10% Solutol HS-15 / 90% PEG 600 via oral gavage. Blood samples were collected after 30 min, 1 h and 3 h (Fig 8). Maximum attainable concentrations via this route of administration were not higher than via the medicated gel. Extrapolation of the time-concentration curve obtained for oral gavage reveals the need for a dosing frequency of at least three times per day in order to obtain sustained levels above the projected EC_50_ of the compound.

## Discussion

4-Fluoro-3’,4’,5’-trimethoxychalcone or C16 is a small molecule with nanomolar anti-invasive activity, a defined structure-activity relationship and a satisfactory ADME and toxicity profile *in vitro* [5–9]. In order to examine its potential as a pharmacological tool, prior exploration of its *in vivo* pharmacokinetic profile, toxicity and tolerability was required. The obtained data is also relevant to several other members of the chalcone chemical class, which have been attributed biological activities (mostly at elevated concentrations) [11], but for which associated DMPK profiling is lacking.

### Vehicle development

DMSO is an excellent solvent for C16 but its applicability *in vivo* is limited. Other formulations were therefore evaluated. For low single dose studies, DMSO/Cremophor/water was identified as a suitable vehicle, while for high single-dose evaluation, Solutol HS-15 / PEG 600 proved appropriate. C16 is stable when dissolved in Solutol HS-15 / PEG 600, and the vehicle is well tolerated in the mouse when given *via* oral gavage. It however generated moderate adverse effects on IP administration, which prohibits its IP use in chronic dosing studies.

The suboptimal DMPK properties of C16 (*see* below) would necessitate at least three oral gavage administrations per day to maintain the desired plasma levels in chronic experiments. We therefore turned to self-medication, as this reduces animal handling and stress, and does not disrupt the diurnal rhythm of the animals. It also reduces the burden on the investigator significantly. Administration of C16 via regular drinking water was not possible, as the compound crashes out of solution and moreover is unstable in an aqueous environment upon prolonged dissolution. At the start of this study, a limited number of reports on the use of medicated gels as a route of administration were available. Medicated gels have advantages over mixing in the feed, as new batches can be prepared on a flexible basis and without the need for special equipment. Daily gel intake is comparable to that of regular drinking water, the gel can thus serve as a complete replacement of drinking water over prolonged periods of time. A major aim of this study was the development of a C16-doped gel as a vehicle for chronic dosing. A suitable formulation was developed in the form of a stable suspension of C16 in Medigel Sucralose. The compound proved stable in this preparation over 144 h, and was shown to be uniformly spread. Earlier reports had validated the tolerability of Medigel Sucralose in rodents. Validation of C16-doped Medigel Sucralose for use in mice was conducted in a repeat-dose toxicity study (described below).

### Pharmacokinetics

The IV, IP and PO pharmacokinetic profile of C16 was determined in rat plasma and mouse whole blood. Regardless of the vehicle (DMSO or DMSO/Cremophor/water), sampling (plasma or whole blood) and species (rat or mouse), the compound shows low (PO) to moderate (IP) bioavailability. In both cases, slow dissolution is a limiting factor, resulting in a similar t_max_ for IP and PO dosing, prolonged exposure and rather flat (rat) or multiple peak (mouse) concentration-time profiles on PO dosing. The use of cassette dosing may have accentuated this phenomenon, though the main cause can be found in the physicochemical profile of C16. It should be noted that the significance of the average t_max_ for PO dosing in rats is limited, as a flat average concentration-time profile was obtained. Despite its good permeability, the low A→B recovery of C16 in the Caco-2 assay suggests cellular metabolism, which also limits oral bioavailability. While the high extent of protein binding may also necessitate elevated oral dosing, the high recovery levels suggest reversible binding.

Regardless of the sampling mode (plasma or whole blood), both rat and mouse half-life on IV dosing are short. This is in line with data published for other chalcones [13]. Nevertheless, an important difference in initially attained IV concentrations was noted, which can be attributed to species and sampling factors. Clearance in both species is much higher than hepatic blood flow (rat: 3.3 L/h/kg, mouse: 5.4 L/h/kg), which points towards extra-hepatic elimination. C16 shows high apparent blood partitioning and a large difference between plasma and whole blood half-life. Rather than irreversible binding to erythrocytes, the compound was found to be metabolized rapidly with glutathione conjugation as the major mechanism. Erythrocytes are a major site of biotransformation and contain cytosolic glutathione S-transferases [21]. The conjugates are then actively transported outward across the red-cell membrane [22]. Metabolism in red blood cells thus seems the major route of extra-hepatic elimination for C16, explaining its much shorter half-life in blood versus plasma. This route of metabolism was not observed for other rapidly metabolized chalcones such as cardamonin [14]. As a further consequence, the higher reported blood-to-plasma ratio is not accurate because it was not based on measurements of both blood and plasma fractions.

### Tolerability, toxicity and chronic systemic availability

The maximum tolerated single dose of C16 in the mouse on administration via oral gavage is >300 mg/kg. The compound was well tolerated, and induced only mild behavioral effects at 100 and 300 mg/kg (vocalization and increased sensitivity to touch). The pre-defined humane end points (*see* Methods *section*) were not reached. No other treatment-induced phenomena were noted on observation or necropsy. The animals proved much more sensitive to intraperitoneal delivery of a single dose of the same vehicle and compound, though humane end points were again not reached. These observations can be correlated to a low tolerability of the vehicle itself, and the earlier observed higher C_max_ for C16 on IP delivery with respect to oral administration (Table 2). Moderate to severe behavioral, neurologic and autonomic effects were noted in a dose-response pattern. These results indicate that the vehicle and C16 up to 50 mg/kg may be tolerated for single-dose IP administration in the mouse, but not for chronic experiments.

Despite its low oral bioavailability, the high oral tolerability of C16 offers opportunities to obtain sufficiently high systemic levels for efficacy testing. This hypothesis was tested in a repeat-dose experiment. The 7-day continuous dosing experiment validated Medigel sucralose as a vehicle for self-medication of C16 in a chronic setting. Substitution of drinking water for blank gels caused a slight but non-significant decrease in weight during the acclimatization period due to neophobia. During the further course of the experiment, however, treatment with solvent-doped gels (DMSO) did not generate significant differences in weight, water and food intake, and hematological and biochemical parameters with respect to the water cohort. No gross lesions or differences in organ weights were noted on necropsy. A slight hepatocyte toxicity, however, was noted for some gel-treated animals, which was absent in the water cohort. This effect may be due to the relatively high DMSO content of the gel. DMSO hepatotoxicity has been reported in chronic trials in laboratory animals [23–26]. In our setting the effect was not major, nor is it reflected in the clinical chemistry panel. The DMSO dose was also well below the LD_50_ in the mouse (21–28 g/kg, PO) [25,27]. Overall, Medigel Sucralose thus proved a suitable vehicle for chronic studies.

Oral C16 dosing via medicated gels over a 7-day period caused sustained systemic levels above its projected EC_50_ value (6.32 ng/mL). This is a significant result, as a major reason for poor correlation between *in vitro* and *in vivo* efficacy of chalcones has been their poor pharmacokinetics and bioavailability, yielding plasma levels below the EC_50_ of the compounds [18]. Gel intake was close to the anticipated amount for all cohorts, resulting in an actual daily oral dose close to the intended. C16 showed nonlinear uptake as systemic levels did not differ significantly between the 100 mg/kg and 300 mg/kg cohort. Given the physicochemical profile of C16, this is likely due to limits in dissolution. Lower dosing regimens may thus still generate sufficient systemic levels, but were not evaluated in this work. Importantly, as expected from the low-dose PK studies, the high-dose concentration-time profile obtained after a single administration of 300 mg/kg C16 via oral gavage confirms the need for TID dosing in order to reach sustained levels above the EC_50_. This underpins the value of the developed medicated gel formulation in the present research and for others working on related molecules.

The confirmed seven-day exposure to the molecule at levels above its EC_50_ did not engender treatment-associated differences between the 0, 100 and 300 mg/kg cohorts, except for a slightly lower kidney weight in the 300 mg/kg cohort. Nephrotoxicity was however not detected in any of the cohorts during histological examination. Hematological parameters were all satisfactory. Some individual measurements fall slightly outside of reported reference ranges, but these ranges itself vary strongly upon the cited source and underlying animal-related variables. The only valid comparison of therapy response thus is to the age-, strain-, and sex-matched controls of the water cohort [28]. No differences were found, except for a lower average glucose reading in the water cohort due to fasting prior to sampling.

Oral delivery using our medicated gel formulation thus is a suitable tool for evaluating the efficacy of C16 in a range of invasion and metastasis models. Depending on the models and more specifically on the interrogated phenomena (local invasion, regional or distal metastasis), compatible dosing schedules may include prophylactic treatment over a 7-day period before inoculation (e.g. in a peritoneal metastasis model), intermittent or low-dose chronic treatment in long-term experiments (months) or high-dose treatment in short-term, more aggressive models (weeks) [29–31]. From the data gathered in this report, adequate exposure and good tolerability of C16 can be expected in these settings.

## Conclusions

C16, like other members of the chalcone family [13,14], shows suboptimal pharmacokinetics in the rat and the mouse. Its limited solubility and cellular metabolism causes poor intestinal uptake and limited oral bioavailability. The compound is rapidly metabolized, with a clearance rate exceeding hepatic blood flow. Glutathione conjugation in erythrocytes was identified as a major route of extra-hepatic elimination, explaining the much shorter half-life of C16 in blood versus plasma. An oral dosing routine using medicated gels was developed that solved these issues. A schedule providing amounts equivalent to oral gavage at 100 mg/kg three times per day yielded sustained whole blood levels above the EC_50_ in a 7-day chronic study. The compound showed good tolerability during the latter experiment. Evaluation of C16 as a pharmacological tool in animal models of metastatic disease, using the medicated gel formulation, is currently ongoing. Besides, we are preparing analogs with a superior DMPK profile in search of improved clinical relevance.

## Supporting information

S1 File This file contains detailed materials and methods, raw data and additional information on selected experiments. (PDF)

## Abbreviations

ACN: acetonitrile
ADME: absorption, distribution, metabolism, excretion
AUC: area under the time-concentration curve
AUMC: area under the moment curve
C16: 4-fluoro-3’,4’,5’-trimethoxychalcone
CL: clearance
DMA: N,N-dimethylacetamide
DMPK: drug metabolism and pharmacokinetics
DMSO: dimethyl sulfoxide
EC_50_: half maximal effective concentration
EDTA: ethylenediaminetetraacetic acid
HPLC-MS/MS: high-performance liquid chromatography-mass spectrometry/mass spectrometry
IP: intraperitoneal
IS: internal standard
IV: intravenous
JVC: jugular vein cannula
MRM: multiple reaction monitoring
MRT: mean residence time
MTD: maximum tolerated dose
NMR: nuclear magnetic resonance
PEG: polyethylene glycol
PG: propylene glycol
PK: pharmacokinetics
PO: *per os*
SAR: structure-activity relationship
TID: *ter in die*
V_ss_: steady-state volume of distribution
