# Supplementary material for: Vehicle development, pharmacokinetics and toxicity of the anti-invasive agent 4-fluoro-3’,4’,5’-trimethoxychalcone in rodents
Source: PLoS One. 2018 Feb 22;13(2):e0192548. doi: 10.1371/journal.pone.0192548 (PMC5823406; doi:10.1371/journal.pone.0192548)
Supplement: S1 File — (PDF) [file pone.0192548.s001.pdf]

S1 File

Vehicle development, pharmacokinetics and toxicity of the anti-invasive agent 4-fluoro-3',4',5'-trimethoxychalcone in rodents

Liselot M. Mus[1,3], Geertrui Denecker[1,3], Frank Speleman[1,3], Bart I. Roman[2,3,]*

[1] Center for Medical Genetics, Ghent University Hospital, De Pintelaan 185, 9000 Ghent, Belgium.

[2] SynBioC Research Group, Department of Green Chemistry and Technology, Campus Coupure, Ghent University, Coupure Links 653, 9000 Ghent, Belgium.

[3] Cancer Research Institute Ghent (CRIG), 9000 Ghent, Belgium.

[*] Corresponding author

E-mail: bart1.roman@ugent.be, bart.roman@gmail.com (BIR)

Contents

A. Vehicle development 2
- A.A. Vehicles for single-dose studies 2
  - *a. Stability test of C16 in 10% Solutol HS -15 / 90% PEG 600 .... 2*
- A.B. Vehicle development for repeated high-dose PO administration of C16 2
  - *b. Stability test of the 300 mg/kg preparation of C16 in Medigel Sucralose at room temperature 2*

B. PK studies 4
- B.A. Rat plasma PK 4
  - *a. Dosing solution preparation 4*
  - *b. Results 4*
- B.B. Mouse blood PK 6
  - *a. Solution preparation 6*
  - *b. Data 6*

C. Stability and metabolism 9
- C.A. Whole blood stability 9
  - *a. Protocol 9*
  - *b. Results 9*

D. Single-dose MTD study 10
- D.A. PO administration 10
- D.B. IP Administration 12

E. Repeat-dose toxicity study 14
- E.A. Tribromoethanol injectable preparation 14
- E.B. Toxicology 14
- E.C. Whole blood C16 levels 17

F. References 17

A. Vehicle development

A.A. Vehicles for single-dose studies

a. *Stability test of C16 in 10% Solutol HS -15 / 90% PEG 600*

Analyte:	C16
Q1/Q3 Masses:	317.1/149.0
IS:	Oxybutynin
Q1/Q3 Masses:	358.5/141.9

Quantification was performed by calculating the ratio of the peak area (counts) of C16 and the IS. Results are expressed as relative differences to the peak ratio at 0 h (%RE, Table A).

Table A. Stability in 10% Solutol HS -15 / 90% PEG 600: peak ratios.

	0 h	1 h	2 h	4 h	8 h	24 h
**10mg/mL at room temperature**						
1	0.513	0.481	0.488	0.578	0.509	0.529
2	0.481	0.494	0.521	0.444	0.490	0.524
3	0.476	0.522	0.489	0.432	0.490	0.459
Mean	0.490	0.499	0.499	0.485	0.497	0.504
SD	0.020	0.021	0.019	0.081	0.011	0.039
%CV	4.1	4.2	3.8	16.7	2.2	7.7
**%RE to 0 h**		**1.8**	**1.8**	**-1.0**	**1.4**	**2.9**
**10mg/mL at 4 °C**						
1	0.513	0.475	0.468	0.519	0.507	0.465
2	0.481	0.461	0.499	0.496	0.463	0.478
3	0.476	0.553	0.461	0.475	0.491	0.506
Mean	0.490	0.496	0.476	0.497	0.487	0.483
SD	0.020	0.049	0.020	0.022	0.022	0.021
%CV	4.1	9.9	4.2	4.4	4.5	4.3
**%RE to 0 h**		**1.2**	**-2.9**	**1.4**	**-0.6**	**-1.4**
**30mg/mL at room temperature**						
1	1.465	1.457	1.418	1.246	1.242	1.570
2	1.468	1.474	1.431	1.586	1.426	1.407
3	1.472	1.446	1.563	1.437	1.495	1.440
Mean	1.468	1.459	1.471	1.423	1.388	1.472
SD	0.003	0.014	0.08	0.171	0.131	0.086
%CV	0.2	1.0	5.4	12.0	9.4	5.8
**%RE to 0 h**		**-0.6**	**0.2**	**-3.1**	**-5.4**	**0.3**
**30mg/mL at 4°C**						
1	1.465	1.840	1.353	1.687	1.680	1.416
2	1.468	1.322	1.444	1.469	1.360	1.532
3	1.472	1.452	1.340	1.405	1.586	1.527
Mean	1.468	1.538	1.379	1.52	1.542	1.492
SD	0.003	0.269	0.057	0.148	0.164	0.065
%CV	0.2	17.5	4.1	9.7	10.6	4.4
**%RE to 0 h**		**4.8**	**-6.1**	**3.5**	**5.0**	**1.6**

CV: Coefficient of variation; RE: Difference in peak area relative to indicated time point.

A.B. Vehicle development for repeated high-dose PO administration of C16

b. *Stability test of the 300 mg/kg preparation of C16 in Medigel Sucralose at room temperature*

The 300 mg/kg preparation refers to a cup containing 235.29 mg of C16 and 1.64% DMSO (*see* Materials and Methods section of main text). Sampling took place for a solvent control cup (containing only 1.64% DMSO), and for the medicated cup at 0.17, 5.33, 55, 102 and 144 h post preparation. Samples of 1 mL were withdrawn with syringe and needle through the lid, and mixed with 1 mL of acetonitrile. The supernatant of the resulting suspension was filtered through a Whatman 0.2 µm PTFE syringe filter and analyzed using HPLC-MS.

HPLC conditions

Instrument:	Agilent 1200 series HPLC system
Column:	Ascentis Express C18 column 2.7 µm (30 x 4.6 mm)
Mobile phase A:	Water (H_2O) + 5 mM NH_4OAc
Mobile phase B:	Acetonitrile
Column Temperature:	40 °C
Injection Volume:	2 µL
UV Detector:	Diode array detector, 190 – 400 nm

See Table B.

Table B. Stability of C16 in Medigel Sucralose: gradient program.

**Time** (min)	**Flow rate** (mL/min)	**A** (%)	**B** (%)
0.00	1	70.0	30.0
0.60	1	70.0	30.0
3.00	1	0	100.0
3.60	1	0	100.0
4.20	1	70.0	30.0
4.80	1	70.0	30.0

Mass spectrometer conditions

Instrument:	Agilent 1100 series VL mass spectrometer
Ionization mode:	API-ES, positive ions

Raw data

Analyte name:	C16
Retention time:	3.00 min
Mass:	317.2
IS:	DMSO
Retention time:	0.31 min
Main derivative:	*Z*-isomer of C16
Retention time:	3.34 min
Mass:	317.1

See Table C.

Table C. Stability of C16 in Medigel Sucralose: evolution of peak areas of IS, C16 and its *Z*-isomer in time for three wavelengths.

**Sample** time (h)	**220.8 nm**				**254.8 nm**				**280.8 nm**			
	**IS peak area** (mAU.s)	**C16 peak area** (mAU.s)	**Z-isomer peak area** (mAU.s)	**Ratio (C16/IS peak area)**	**IS peak area** (mAU.s)	**C16 peak area** (mAU.s)	**Z-isomer peak area** (mAU.s)	**Ratio (C16/IS peak area)**	**IS peak area** (mAU.s)	**C16 peak area** (mAU.s)	**Z-isomer peak area** (mAU.s)	**Ratio (C16/IS peak area)**
Solvent control	5109	0	0	0.000	4910	0	0	0.000	830	0	0	0.000
0.17	7815	1638	0	0.210	4542	958	0	0.211	754	149	0	0.198
5.33	8000	1662	169	0.208	5255	920		0.175	855	150	76	0.176
55	7889	1489	481	0.189	4602	872		0.189	796	166	169	0.208
102	8477	1675	181	0.198	4959	973	11	0.196	811	166	71	0.205
144	8198	1563	598	0.191	4743	865	58	0.182	784	124	191	0.158

Peak ratios for stability assessment

Peak ratios for C16/IS, relative to the first time point, were calculated for three wavelengths (Table D).

Table D. Stability of C16 in Medigel Sucralose: peak ratios for C16/IS, relative to first time point, for three wavelengths.

**Time** (h)	**220.8 nm**	**254.8 nm**	**280.8 nm**	**Average**
0.17	100	100	100	100
5.3	99.1	83.0	88.7	90.3
55	90.0	89.8	105.2	95.0
102	94.3	93.0	103.5	96.9
144	91.0	86.5	80.0	85.8

Peak ratios for homogeneity assessment of a 300 mg/kg C16-doped gel

The 300 mg/kg preparation refers to a cup containing 235.29 mg of C16 and 1.64% DMSO. After preparation (*see 'Materials and Methods' section of main text*), the gel was sampled through the lid at five sites (Fig A).

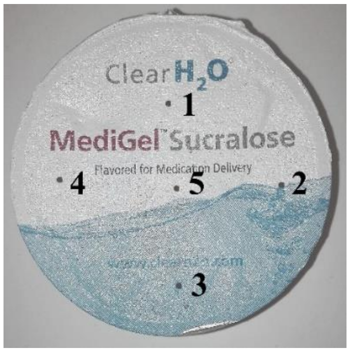

Fig A. Sampling of C16-doped Medigel Sucralose.

The samples were analyzed according to the method described above for the stability test of C16 in Medigel Sucralose (Table E).

Table E. Peak areas of IS, C16 and its *Z*-isomer at five sampling sites for three wavelengths.

**Sampling sites**	**220.8 nm**			**254.8 nm**			**280.8 nm**		
	**IS peak area** (mAU.s)	**C16 peak area** (mAU.s)	**Ratio (C16/IS peak area)**	**IS peak area** (mAU.s)	**C16 peak area** (mAU.s)	**Ratio (C16/IS peak area)**	**IS peak area** (mAU.s)	**C16 peak area** (mAU.s)	**Ratio (C16/IS peak area)**
1	8495	1365	0.16	5245	754	0.14	826	105	0.13
2	8255	1279	0.15	4928	593	0.12	835	110	0.13
3	8519	1593	0.19	5513	830	0.15	898	136	0.15
4	8439	1622	0.19	5390	796	0.15	820	139	0.17
5	8409	1596	0.19	5293	783	0.15	819	126	0.15
**Average±SD**			**0.18±0.02**			**0.14±0.01**			**0.15±0.02**

B. PK studies

B.A. Rat plasma PK

a. Dosing solution preparation

The dosing solution was prepared as a cassette to contain all three test articles in the same solution. IV dosing was fixed at 2 mg/mL of C16 and the two other test articles in neat DMSO as the vehicle, with a dosing volume 0.5 mL/kg (slow injection). PO dosing was set at 10 mg/mL of C16 and the two other test articles in neat DMSO as the vehicle, with a dosing volume of 1 mL/kg.

b. Results

Individual plasma concentrations for C16 are shown in Table F and Table G. All data are expressed as ng/mL of the free drug. Samples that were below the limit of quantitation were not used in the calculation of averages. Plasma concentrations versus time data are plotted in Fig B and Fig C. Measured dosing concentrations were used in all pharmacokinetic calculations.

Table F. Individual plasma concentrations (ng/mL) and pharmacokinetic parameters for C16 after intravenous administration in male Sprague-Dawley rats at 1 mg/kg (in 100% DMSO).

**Parameter**		**Animal**		
		**1**	**2**	**3**
t (h)	0 (pre-dose)	BLOQ	BLOQ	BLOQ
	0.083	568	345	693
	0.25	62.1	94.6	171
	0.50	32.4	31.9	63.2
	1.0	23.1	19.1	25.4
	2.0	**14.3**	**8.99**	**12.6**
	4.0	**4.94**	**4.34**	**6.41**
	8.0	**1.34**	**1.12**	**1.58**
Animal weight (kg)		0.324	0.332	0.311
Amount dosed (mL)		0.32	0.33	0.31
c_0 (ng/mL) [a]		1718	659	1395
t_{max} (h) [a]		0.0	0.0	0.0
$t_{1/2}$ (h)		1.80	2.00	2.00
MRT_{last} (h)		0.70	0.87	0.71
CL (L/h/kg)		4.40	6.73	3.72
V_{ss} (L/kg)		3.73	7.30	3.27
AUC_{last} (h·ng/mL)		224	145	264
AUC_{∞} (h·ng/mL)		227	149	269

c_0: Maximum plasma concentration extrapolated to t =0; t_{max}: Time of maximum plasma concentration; $t_{1/2}$: Half-life, data points used for half-life determination are in bold; MRT_{last}: Mean residence time, calculated to the last observable time point; CL: Clearance; V_{ss}: Steady state volume of distribution; AUC_{last}: Area under the curve, calculated to the last observable time point; AUC_{∞}: Area under the curve, extrapolated to infinity; ND: Not determined; BLOQ: Below the limit of quantitation (1 ng/mL); [a] Extrapolated to t =0.

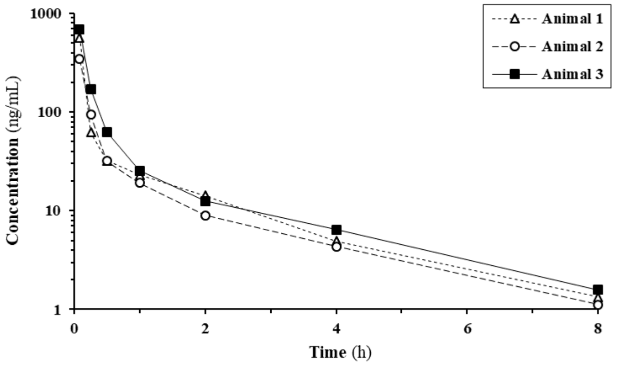

Fig B. Individual plasma concentration-time curves of C16 on IV administration in male Sprague-Dawley rats at 1 mg/kg in 100% DMSO.

Table G. Individual plasma concentrations (ng/mL) and pharmacokinetic parameters for C16 after oral administration in male Sprague-Dawley rats at 10 mg/kg (in 100% DMSO).

**Parameter**		**Animal**		
		**4**	**5**	**6**
t (h)	0 (pre-dose)	BLOQ	BLOQ	BLOQ
	0.25	21.8	15.9	33.5
	0.50	22.5	14.8	24.0
	1.0	28.3	13.6	22.0
	2.0	**15.1**	**11.4**	**18.2**
	4.0	**27.9**	**11.7**	**13.6**
	8.0	**47.1**	**8.04**	**5.96**
Animal weight (kg)		0.326	0.329	0.320
Amount dosed (mL)		0.65	0.66	0.64
c_{max} (ng/mL)		47.1	15.9	33.5
t_{max} (h)		8.00	0.25	0.25
$t_{1/2}$ (h)		ND [a]	11.0	3.67
MRT_{last} (h)		4.92	3.60	2.48

Table G (*continued*). Individual plasma concentrations (ng/mL) and pharmacokinetic parameters for C16 after oral administration in male Sprague-Dawley rats at 10 mg/kg (in 100% DMSO).

Parameter	Animal		
	**4**	**5**	**6**
AUC_{last} (h·ng/mL)	236	88.0	114
AUC_{∞} (h·ng/mL)	ND [a]	219	102
**Dose Normalized Values**			
AUC_{last} (h·kg·ng/mL/mg) [b]	23.6	8.80	11.4
AUC_{∞} (h·kg·ng/mL/mg) [b]	ND [a]	21.9	10.2
Bioavailability (%) [c]	11.2	4.17	5.39

c_{max}: Maximum plasma concentration; t_{max}: Time of maximum plasma concentration; $t_{1/2}$: Half-life, data points used for half-life determination are in bold; MRT_{last}: Mean residence time, calculated to the last observable time point; AUC_{last}: Area under the curve, calculated to the last observable time point; AUC_{∞}: Area under the curve, extrapolated to infinity; ND: Not determined; BLOQ: Below the limit of quantitation (1 ng/mL); [a] Not determined due to correlation coefficient (R^2) was less than 0.85; [b] Dose normalized by dividing the parameter by the nominal dose in mg/kg; [c] Bioavailability determined by dividing the individual dose normalized oral AUC_{last} by the average dose normalized intravenous AUC_{last} value.

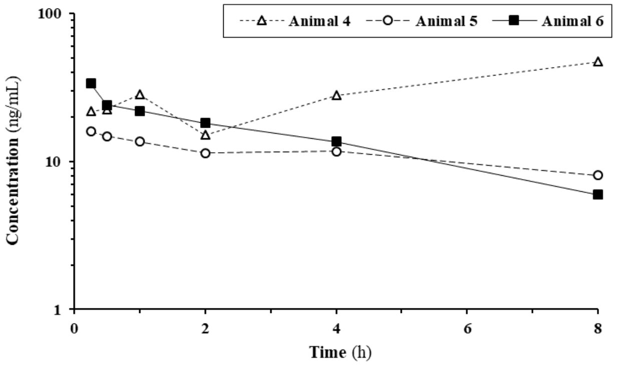

Fig C. Individual plasma concentration-time curves of C16 following PO in male Sprague-Dawley rats at 10 mg/kg in 100% DMSO.

B.B. Mouse blood PK

a. *Solution preparation*

The dosing solution was prepared as a cassette to contain all three test articles in the same solutions using a vehicle consisting of 20% DMSO/10% (DMSO/Cremophor EL 1:1)/70% Milli-Q water.

b. *Data*

Individual blood concentrations and pharmacokinetic parameters are shown in Table H through Table J. All data are expressed as ng/mL of the free drug. Samples that were below the limit of quantification were not used in the calculation of averages. Concentration versus time data are plotted in Fig D through Fig F.

Table H. Individual blood concentrations (ng/mL) and PK parameters for C16 after IV administration in male CD-1 mice at 1 mg/kg.

		Animal		
		**1**	**2**	**3**
t (h)	0 (pre-dose)	BLOQ	BLOQ	BLOQ
	0.083	46.4	54.4	33.4
	0.25	17.3	15.2	**15.4**
	0.50	**8.40**	**8.57**	**10.9**
	1.0	**4.50**	**4.93**	**3.76**
	2.0	**2.28**	**2.21**	**2.30**
	4.0	BLOQ	BLOQ	BLOQ
	8.0	BLOQ	BLOQ	BLOQ

Table H (*continued*). Individual blood concentrations (ng/mL) and PK parameters for C16 after IV administration in male CD-1 mice at 1 mg/kg.

	Animal		
	**1**	**2**	**3**
Animal weight (kg)	0.032	0.032	0.035
Volume dosed (mL)	0.064	0.064	0.070
c_0 (ng/mL) [a]	75.8	103	49.1
t_{max} (h) [a]	0	0	0
$t_{1/2}$ (h)	0.823	0.780	0.636
MRT_{last} (h)	0.426	0.399	0.476
CL (L/h/kg)	43.6	40.4	51.0
V_{ss} (L/kg)	32.8	27.2	37.7
AUC_{last} (h·ng/mL)	20.2	22.2	17.5
AUC_{∞} (h·ng/mL)	22.9	24.7	19.6

C_0: Maximum blood concentration extrapolated to t =0; t_{max}: Time of maximum blood concentration; $t_{1/2}$: Half-life, data points used for half-life determination are in bold; MRT_{last}: Mean residence time, calculated to the last observable time point; CL: Clearance; V_{ss}: Steady state volume of distribution; AUC_{last}: Area under the curve, calculated to the last observable time point; AUC_{∞}: Area under the curve, extrapolated to infinity; ND: Not determined; BLOQ: Below the limit of quantitation (1 ng/mL); [a] Extrapolated to t =0.

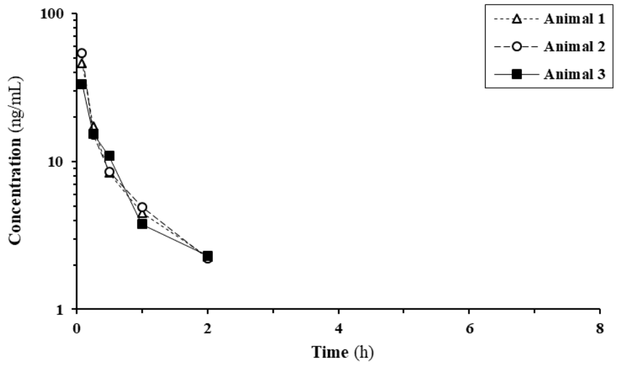

Fig D. Individual blood concentration-time curves of C16 following IV administration in Male CD-1 mice at 1 mg/kg.

Table I. Individual blood concentrations (ng/mL) and PK parameters for C16 after IP administration in male CD-1 mice at 10 mg/kg.

		Animal		
		**4**	**5**	**6**
t (h)	0 (pre-dose)	BLOQ	BLOQ	BLOQ
	0.083	28.2	68.6	64.5
	0.25	90.3	106	125
	0.50	48.2	**94.4**	**56.3**
	1.0	**49.2**	**48.0**	**32.1**
	2.0	**13.4**	**13.3**	**12.1**
	4.0	**1.79**	**5.29**	**3.62**
	8.0	BLOQ	BLOQ	BLOQ
Animal weight (kg)		0.026	0.028	0.026
Volume dosed (mL)		0.13	0.14	0.13
c_{max} (ng/mL)		90.3	106	125
t_{max} (h)		0.25	0.25	0.25
$t_{1/2}$ (h)		0.636	0.862	0.901
MRT_{last} (h)		0.991	0.950	0.916
AUC_{last} (h·ng/mL)		99.2	127	101
AUC_{∞} (h·ng/mL)		101	134	106
**Dose-normalized values** [a]				
AUC_{last} (h·kg ng/mL/mg)		9.92	12.7	10.1
AUC_{∞} (h·kg ng/mL/mg)		10.1	13.4	10.6
Bioavailability (%) [b]		49.7	63.7	50.6

c_{max}: Maximum blood concentration; t_{max}: Time of maximum blood concentration; $t_{1/2}$: Half-life, data points used for half-life determination are in bold; MRT_{last}: Mean residence time, calculated to the last observable time point; AUC_{last}: Area under the curve, calculated to the last observable time point; AUC_{∞}: Area under the curve, extrapolated to infinity; ND: Not determined; BLOQ: Below the limit of quantitation (1 ng/mL); [a] Dose-normalized by dividing the parameter by the nominal dose of 10 mg/kg; [b] Bioavailability determined by dividing the individual dose-normalized IP AUC_{last} values by the average dose-normalized IV AUC_{last} value.

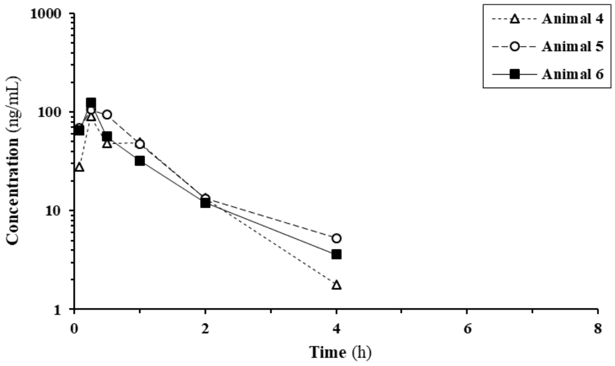

Fig E. Individual blood concentration-time curves of C16 following IP administration in male CD-1 mice at 10 mg/kg.

Table J. Individual blood concentrations (ng/mL) and PK parameters for C16 after PO administration in male CD-1 mice at 10 mg/kg.

		**Animal**		
		**7**	**8**	**9**
t (h)	0 (pre-dose)	BLOQ	BLOQ	BLOQ
	0.25	8.76	17.8	10.9
	0.50	**7.95**	12.4	5.89
	1.0	**7.01**	5.15	3.45
	2.0	**6.19**	4.28	10.5
	4.0	**3.44**	BLOQ	1.26
	8.0	BLOQ	BLOQ	BLOQ
Animal weight (kg)		0.028	0.027	0.028
Volume dosed (mL)		0.14	0.14	0.14
c_{max} (ng/mL)		8.76	17.8	10.9
t_{max} (h)		0.25	0.25	0.25
$t_{1/2}$ (h)		2.93	ND [d]	ND [d]
MRT_{last} (h)		1.71	0.767	1.67
AUC_{last} (h·ng/mL)		23.2	15.1	24.5
AUC_{∞} (h·ng/mL)		ND [c]	ND [d]	ND [d]
**Dose-normalized values** [a]				
AUC_{last} (h·kg ng/mL/mg)		2.32	1.51	2.45
AUC_{∞} (h·kg ng/mL/mg)		ND [c]	ND [d]	ND [d]
Bioavailability (%) [b]		11.6	7.56	12.3

c_{max}: Maximum blood concentration; t_{max}: Time of maximum blood concentration; $t_{1/2}$: Half-life, data points used for half-life determination are in bold; MRT_{last}: Mean residence time, calculated to the last observable time point; AUC_{last}: Area under the curve, calculated to the last observable time point; AUC_{∞}: Area under the curve, extrapolated to infinity; ND: Not determined; BLOQ: Below the limit of quantitation (1 ng/mL); [a] Dose-normalized by dividing the parameter by the nominal dose of 10 mg/kg; [b] Bioavailability determined by dividing the individual dose-normalized oral AUC_{last} values by the average dose-normalized IV AUC_{last} value; [c] Not determined because the AUC was a greater than 25% extrapolation above the AUClast value; [d] Not determined because the line defining the terminal elimination phase had an r^2 of <0.85.

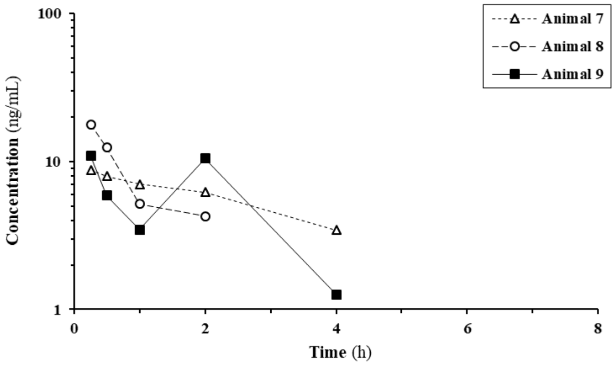

Fig F. Individual blood concentration-time curves of C16 following PO administration in male CD-1 mice at 10 mg/kg.

C. Stability and metabolism

For details on ADME experiments not included underneath, please consult ref. [1].

C.A. Whole blood stability

a. Protocol

HPLC conditions

Column: Kinetex XB-C18 column, 2.6 µm (150 x2.1 mm)
Mobile phase A: H_2O + 0.1% v/v Formic Acid
Mobile phase B: MeCN + 0.1% v/v Formic Acid
Temperature: 60°C
Autosampler syringe volume: 100 µL
Loop volume: 25 µL
Injection volume: 10 µL
Weak wash: MeOH:H_2O 1:9 v/v
Strong wash: MeOH:H_2O 95:5 v/v
See Table K.

Table K. Gradient program.

**Time** (min)	**Flow rate** (mL/min)	**A (%)**	**B (%)**
0.00	0.4	98	2
0.50	0.4	98	2
10.00	0.4	14	86
11.00	0.4	5	95
12.00	0.4	5	95
12.50	0.4	98	2
15.00	0.4	98	2

Mass spectrometric conditions

Model: AB Sciex API5500 QTrap
Mode: Multiple ion monitoring (MIM)
See Table L.

Table L. MIM (parent to parent transitions).

**Analyte**	**Q1** (Da)	**Q3** (Da)	**Dwell** (msec)
C16	317.2	317.2	20
Z-isomer	317.2	317.2	20
Potential glutathione conjugate	624.3	624.3	20

b. Results

The areas and percentages for the parent and metabolites have been calculated using MIM data where parent to parent transitions have been used, and assume that each metabolite has the same point of ionisation and the sensitivity of the metabolite has not been affected by the biotransformation. All remaining peaks in the sample were either in the control sample or not believed to be related to test compound. *See* Table M through Table P.

Table M. Stability of C16 in male Sprague-Dawley rat blood.

**Compound**	**Compound remaining (% of 0 min)**					**Comments**
	**0 min**	**15 min**	**30 min**	**60 min**	**120 min**	
C16	100	16.5	13.8	10.6	7.00	Second peak observed, *Z*-isomer of C16 (peak included).
diltiazem	100	99.9	91.1	74.3	45.5	Reference

Table N. Intrinsic clearance and half-life of C16 in male Sprague-Dawley rat blood.

**Compound**		**Retention time** (min)	**Intrinsic clearance** (µL/min/mL blood)	***n***	$t_{1/2}$ (min)	**Comments**
C16	Both Isomers	Both integrated	120	2	5,76	30, 60 and 120 min excluded.
	E-isomer only	1,76	86,0	2	8,06	30, 60 and 120 min excluded.
	Z-isomer only	1,66	190	2	3,65	30, 60 and 120 min excluded.

Table O. Summary of metabolites of C16 in male Sprague-Dawley rat blood.

Compound	Mass	Metabolite name		Formula	Mass difference from parent	ES+ *m/z* found	Retention time (min)
**C16**	316	Parent				317	8.4
	316	Z-isomer	A			317	8.0
	623	Potential glutathione conjugation	B	+ $C_{10}H_{17}N_3O_6S$	+307	624	5.3

Table P. Summary of parent/metabolite areas and percentage in 0 min control and 15 min human samples.

Compound	Absolute area in sample		Area percentage in sample	
	**0 min control**	**15 min sample**	**0 min control**	**15 min sample**
C16	$1.71e^7$	$5.21e^6$	62.4	52.2
Z-isomer	$1.00e^7$	$1.07e^6$	36.5	10.7
Potential glutathione conjugation	$2.89e^5$	$3.70e^6$	1.06	37.1

D. Single-dose MTD study

D.A. PO administration

C16 was administered orally to groups of 2 male and 2 female ICR mice and the animals were observed for the presence of acute toxic symptoms and autonomic effects during the first 60 min after dosing (Table Q, Table R). The dose was increased or decreased in each subsequent test round until the MTD was discovered. Gross necropsy was performed in all animals without tissue collection.

Table Q. Body weights, evolution over 72 h, PO MTD study.

Compound	Dose	Sex	Animal	Body weight (g)	
				**Pre-dose**	**72 h**
Vehicle	10 mL/kg	M	1-1	24	29
(10% Solutol HS-15 / 90% PEG 600)			1-2	24	29
		F	2-1	23	23
			2-2	23	25
C16	10 mg/kg	M	3-1	22	28
			3-2	22	27
		F	4-1	23	23
			4-2	23	24
	30 mg/kg	M	5-1	25	29
			5-2	25	30
		F	6-1	24	26
			6-2	24	25
	100 mg/kg	M	7-1	24	30
			7-2	25	31
		F	8-1	23	25
			8-2	26	28
	300 mg/kg	M	9-1	23	27
			9-2	23	27
		F	10-1	23	24
			10-2	22	24

Table R. Observations during PO MTD study.

**Treatment**	**Vehicle (10% Solutol HS-15 / 90% PEG 600)**				**C16**															
**Dosage**	**10 mL/kg**				**10 mg/kg**				**30 mg/kg**				**100 mg/kg**				**300 mg/kg**			
**Sex**	**M**		**F**		**M**		**F**		**M**		**F**		**M**		**F**		**M**		**F**	
**Animal**	**1-1**	**1-2**	**2-1**	**2-2**	**3-1**	**3-2**	**4-1**	**4-2**	**5-1**	**5-2**	**6-1**	**6-2**	**7-1**	**7-2**	**8-1**	**8-2**	**9-1**	**9-2**	**10-1**	**10-2**
**Behavioral**																				
B.W. (g)	24	24	25	25	24	24	23	23	25	25	24	24	24	25	23	26	23	23	23	22
Irritability	-	-	-	-	-	-	-	-	-	-	-	-	Voc	-	Voc	Voc	Voc	-	Voc	-
Hyperactivity	-	-	-	-	-	-	-	-	-	-	-	-	-	-	-	-	-	-	-	-
Inc. startle	-	-	-	-	-	-	-	-	-	-	-	-	-	-	-	-	-	-	-	-
Inc. touch	-	-	-	-	-	-	-	-	-	-	-	-	±	-	±	-	-	-	-	-
Dec. startle response	-	-	-	-	-	-	-	-	-	-	-	-	-	-	-	-	-	-	-	-
Dec. touch response	-	-	-	-	-	-	-	-	-	-	-	-	-	-	-	-	-	-	-	-
Inc. exploration	-	-	-	-	-	-	-	-	-	-	-	-	-	-	-	-	-	-	-	-
Dec. exploration	-	-	-	-	-	-	-	-	-	-	-	-	-	-	-	-	-	-	-	-
Pinna	-	-	-	-	-	-	-	-	-	-	-	-	-	-	-	-	-	-	-	-
Placing	-	-	-	-	-	-	-	-	-	-	-	-	-	-	-	-	-	-	-	-
**Neurologic**																				
Tremor	-	-	-	-	-	-	-	-	-	-	-	-	-	-	-	-	-	-	-	-
Dec. spont. activity	-	-	-	-	-	-	-	-	-	-	-	-	-	-	-	-	-	-	-	-
Straub tail	-	-	-	-	-	-	-	-	-	-	-	-	-	-	-	-	-	-	-	-
Reactivity	-	-	-	-	-	-	-	-	-	-	-	-	-	-	-	-	-	-	-	-
Righting	-	-	-	-	-	-	-	-	-	-	-	-	-	-	-	-	-	-	-	-
Ataxia	-	-	-	-	-	-	-	-	-	-	-	-	-	-	-	-	-	-	-	-
Convulsion C.T.C-T	-	-	-	-	-	-	-	-	-	-	-	-	-	-	-	-	-	-	-	-
Low limb post	-	-	-	-	-	-	-	-	-	-	-	-	-	-	-	-	-	-	-	-
Abdominal tone	-	-	-	-	-	-	-	-	-	-	-	-	-	-	-	-	-	-	-	-
Limb tone	-	-	-	-	-	-	-	-	-	-	-	-	-	-	-	-	-	-	-	-
Grip strength	-	-	-	-	-	-	-	-	-	-	-	-	-	-	-	-	-	-	-	-
**Autonomic**																				
Skin color	-	-	-	-	-	-	-	-	-	-	-	-	-	-	-	-	-	-	-	-
Respiration	-	-	-	-	-	-	-	-	-	-	-	-	-	-	-	-	-	-	-	-
Salivation F.V.	-	-	-	-	-	-	-	-	-	-	-	-	-	-	-	-	-	-	-	-
Lacrimation	-	-	-	-	-	-	-	-	-	-	-	-	-	-	-	-	-	-	-	-
Diarrhea	-	-	-	-	-	-	-	-	-	-	-	-	-	-	-	-	-	-	-	-
Body temperature	-	-	-	-	-	-	-	-	-	-	-	-	-	-	-	-	-	-	-	-
Piloerection	-	-	-	-	-	-	-	-	-	-	-	-	-	-	-	-	-	-	-	-
Inc. palpebral size	-	-	-	-	-	-	-	-	-	-	-	-	-	-	-	-	-	-	-	-
Dec. palpebral size	-	-	-	-	-	-	-	-	-	-	-	-	-	-	-	-	-	-	-	-
Others	-	-	-	-	-	-	-	-	-	-	-	-	-	-	-	-	-	-	-	-
Death	-	-	-	-	-	-	-	-	-	-	-	-	-	-	-	-	-	-	-	-

Abbreviations: M: Male; F: Female; -: No effects; ±: Slight; Inc.: Increased; Dec.: Decreased; Spont.: Spontaneous; C: Chronic; T: Tonic; C-T: Chronic-tonic; F.: Fast; D: Depth; Voc: Vocalization.

D.B. IP Administration

C16 was administered IP at an initial dose of 10 mg/kg to a group of 5 ICR derived male mice weighing 22±2 g. The animals were then observed for presence of acute toxic symptoms and autonomic effects during the first 30 min and after 1 h, 2 h, 6 h and 24 h (Table S-Table U). The next dose level was determined based on whether more than 50% animals died within 30 min after dosing. Gross necropsy was performed in all animals without tissue collection.

Table S. Body weights, evolution over 24 h, IP MTD study.

**Compound**	**Dose**	**Animal**	**Body weight** (g)	
			**Pre-dose**	**72 h**
Vehicle (10% Solutol HS-15 / 90% PEG 600)	5 mL/kg	1-1	24	23
		1-2	23	21
		1-3	23	22
		1-4	24	23
		1-5	23	22
C16	10 mg/kg	2-1	25	24
		2-2	25	24
		2-3	23	22
		2-4	26	25
		2-5	23	24
	30 mg/kg	3-1	25	23
		3-2	22	23
		3-3	24	23
		3-4	24	23
		3-5	25	22
	50 mg/kg	4-1	24	22
		4-2	26	24
		4-3	25	23
		4-4	26	24
		4-5	25	22

Table T. Food consumption over 24 h, IP MTD study.

**Compound**	**Dose**	**Animal**	**Food consumption** (g)
Vehicle (10% Solutol HS-15 / 90% PEG 600)	5 mL/kg	1-1	2
		1-2	2
		1-3	3
		1-4	2
		1-5	2
C16	10 mg/kg	2-1	2
		2-2	2
		2-3	3
		2-4	3
		2-5	5
	30 mg/kg	3-1	2
		3-2	5
		3-3	3
		3-4	2
		3-5	2
	50 mg/kg	4-1	3
		4-2	2
		4-3	1
		4-4	2
		4-5	1

Table U. Observations during IP MDT study.

Treatment	Vehicle (10% Solutol HS-15 / 90% PEG 600)					C16														
**Dosage**	**5 mL/kg**					**10 mg/kg**					**30 mg/kg**					**50 mg/kg**				
**Animal**	**1-1**	**1-2**	**1-3**	**1-4**	**1-5**	**2-1**	**2-2**	**2-3**	**2-4**	**2-5**	**3-1**	**3-2**	**3-3**	**3-4**	**3-5**	**4-1**	**4-2**	**4-3**	**4-4**	**4-5**
**Behavioral**																				
Irritability	-	-	-	-	-	-	-	-	-	-	-	-	Voc	-	-	-	-	-	Voc	-
Hyperactivity	-	-	-	-	-	-	-	-	-	-	-	-	-	-	-	-	-	-	-	-
Inc. startle	-	-	-	-	-	-	-	-	-	-	-	-	-	-	-	-	-	-	-	-
Inc. touch	-	-	-	-	-	-	-	-	-	-	-	-	-	-	-	-	-	-	-	-
Dec. startle response	-	-	-	-	-	-	-	-	-	-	-	-	-	-	-	-	-	-	-	-
Dec. touch response	-	±	-	-	-	-	±	±	±	-	±	±	±	±	±	±	±	±	±	±
Inc. exploration	-	-	-	-	-	-	-	-	-	-	-	-	-	-	-	-	-	-	-	-
Dec. exploration	±	±	±	-	-	±	±	-	±	-	-	±	-	-	±	±	-	±	-	±
Pinna	-	-	-	-	-	-	-	-	-	-	±	-	-	±	-	±	-	±	-	-
Placing	-	-	-	-	-	-	-	-	-	-	-	-	-	-	-	-	-	-	-	-
**Neurologic**																				
Tremor	-	-	-	-	Twi	Twi	Twi	-	-	-	Twi	-	-	-	Twi	-	Twi	Twi	Twi	-
Dec. spont. activity	-	-	±	-	-	-	±	±	±	-	±	±	±	±	-	±	±	±	±	±
Straub tail	-	-	-	-	-	-	-	-	-	-	-	-	-	-	-	-	-	-	-	-
Reactivity	-	-	-	-	-	-	-	-	-	-	-	-	-	-	-	-	-	-	-	-
Righting	-	-	-	-	-	-	-	-	-	-	-	-	-	-	-	-	-	-	-	-
Ataxia	±	-	-	±	-	±	±	-	-	-	±	-	-	±	±	-	-	±	-	±
Convulsion C.T.C-T	-	-	-	-	-	-	-	-	-	-	-	-	-	-	-	-	-	-	-	-
Low limb post	-	-	-	-	-	-	-	-	-	-	-	-	-	-	±	-	±	±	-	-
Abdominal tone	+	±	±	±	±	+	+	+	+	±	±	±	+	+	+	±	±	+	±	+
Limb tone	+	±	+	±	+	+	+	+	+	±	±	±	+	+	+	+	+	+	+	+
Grip strength	±	±	±	±	±	±	±	±	±	-	±	-	±	±	+	±	±	±	±	±
**Autonomic**																				
Skin color	-	-	-	-	-	-	-	-	-	-	-	-	-	-	-	-	-	-	-	-
Respiration	D±	D±	D±	-	-	D±	-	D±	D±	-	D±	D±	D±	D±	D±	D±	D±	D±	D±	D±
Salivation F.V.	-	-	-	-	-	-	-	-	-	-	-	-	-	-	-	-	-	-	-	-
Lacrimation	-	-	-	-	-	-	-	-	-	-	-	-	-	-	-	-	-	-	-	-
Diarrhea	-	-	-	-	-	-	-	-	-	-	-	-	-	-	-	-	-	-	-	-
Body temperature	-	-	-	-	-	-	-	-	-	-	-	-	-	-	-	±↓	±↓	±↓	±↓	±↓
Piloerection	-	-	-	-	-	-	-	-	-	-	-	-	-	-	-	-	-	-	-	-
Inc. palpebral size	-	-	-	-	-	-	-	-	-	-	-	-	-	-	-	-	-	-	-	-
Dec. palpebral size	-	-	-	-	-	±	±	-	-	-	±	±	±	+	±	±	±	±	±	±
Others	-	-	-	-	-	H.B.	H.B.	-	H.B.	-	H.B.	H.B.	H.B.	H.B.	H.B.	H.B.	H.B.	H.B.	H.B.	H.B.
Death	-	-	-	-	-	-	-	-	-	-	-	-	-	-	-	-	-	-	-	-

Abbreviations: -: No effects; ±: Slight to moderate effects; +: Severe effects; Twi: Twitches; D: Depth; ↓: Low; H.B.: Hunch back; Inc.: Increased; Dec.: Decreased; Spont.: Spontaneous; C: Chronic; T: Tonic; C-T: Chronic-tonic; D: Depth; Voc: Vocalization.

E. Repeat-dose toxicity study

E.A. Tribromoethanol injectable preparation

An IP injectable solution of the anesthetic tribromoethanol (Avertin) was prepared by dissolving 2.5 grams of 2,2,2-tribromoethanol in 5 mL of 2-methyl-2-butanol under gentle heating (40 °C) and vigorous stirring. Distilled water at neutral pH was added up to a final volume of 200 mL. The resulting solution was filtered through a 0.5 µm Millipore filter and collected in a sterile container. The thus obtained solution contains 12.5 mg/mL of 2,2,2-tribromoethanol and can be administered to mice via IP injection at a dose of 250 mg/kg (0.5 mL for a 25 g animal). Induction is fast (~2 min) and the righting reflex returns after 35-90 min. **IMPORTANT**. Solutions should always be vigorously shaken and homogenized before use. Higher concentrated solutions of 2,2,2-tribromoethanol are irritating and should not be used. It is advisable to aliquot the solution and protect the material from heat and light. The product is stable for ~15 days when refrigerated. Degraded solutions become acidic and toxic and should be discarded (test: add one drop of Congo red to 5 mL of the solution: purple coloration indicates the solution should no longer be used).

E.B. Toxicology

An overview of all details and raw data is presented in Table V-Table Z.

Table V. Animal weights during repeat-dose toxicity study.

**Cohort and animal**		**Weights** (g)										
		**Day -3**	**Day -2** [a]	**Day -1**	**Day 0** [b]	**Day 1**	**Day 2**	**Day 3**	**Day 4**	**Day 5**	**Day 6**	**Day 7**
**0 mg/kg**	1-1	27.9	28.2	27.5	27.3	27.1	28.0	29.0	29.0	29.6	29.6	29.8
	1-2	30.8	30.2	29.8	30.3	30.5	30.6	31.4	31.3	30.8	31.7	31.9
	1-3	33.3	32.1	29.8	31.5	31.3	32.3	32.3	32.2	32.7	32.8	33.1
	1-4	29.1	29.6	28.7	30.4	30.1	30.7	31.0	32.2	32.8	31.9	31.5
	**Average±SD**	**30.3±2.4**	**30.0±1.6**	**28.9±1.1**	**29.9±1.8**	**29.8±1.8**	**30.4±1.8**	**30.9±1.4**	**31.2±1.5**	**31.5±1.5**	**31.5±1.4**	**31.6±1.4**
**100 mg/kg**	2-1	34.0	34.2	31.0	31.6	30.3	31.8	32.9	33.3	32.7	32.3	33.6
	2-2	32.9	32.6	31.9	32.5	32.3	31.9	32.6	34.0	34.7	34.3	33.7
	2-3	30.2	30.6	29.2	29.6	29.0	30.0	30.5	31.5	32.4	32.6	34.1
	2-4	29.1	28.7	28.0	28.2	28.5	28.6	28.6	29.3	29.8	29.4	29.3
	**Average±SD**	**31.5±2.3**	**31.5±2.4**	**30.0±1.7**	**30.4±1.9**	**30.0±1.7**	**30.6±1.6**	**31.2±2.0**	**32.0±2.1**	**32.4±2.0**	**32.1±2.0**	**32.7±2.2**
**300 mg/kg**	3-1	30.3	30.5	28.2	27.9	28.0	28.9	29.3	29.9	29.8	30.1	30.9
	3-2	32.8	33.1	30.7	29.8	29.4	30.2	30.8	32.6	33.0	33.8	33.1
	3-3	29.5	29.6	28.0	28.2	28.7	30.4	29.0	30.3	30.9	30.4	30.4
	3-4	28.4	28.6	26.9	27.4	28.3	29.8	29.3	29.3	29.3	30.1	30.8
	**Average±SD**	**30.2±1.9**	**30.4±2.0**	**28.4±1.6**	**28.3±1.1**	**28.6±0.6**	**29.8±0.7**	**29.6±0.8**	**30.5±1.4**	**30.7±1.6**	**31.1±1.8**	**31.3±1.2**
**Water** [c]	4-1	28.7	28.9	29.2	28.8	28.6	28.8	29.0	29.4	29.4	29.3	28.2
	4-2	29.5	31.0	32.0	31.0	31.1	32.9	32.3	32.9	32.8	32.3	31.4
	4-3	33.1	31.0	30.3	29.4	30.2	30.5	30.6	30.5	31.6	31.3	29.3
	**Average±SD**	**30.4±2.4**	**30.3±1.2**	**30.5±1.4**	**29.7±1.2**	**29.6±1.2**	**30.7±2.1**	**30.6±1.7**	**30.9±1.8**	**31.3±1.7**	**31.0±1.5**	**29.6±1.6**
	p	0.811 [d]	0.710 [e]	0.279 [d]	0.322 [d]	0.493 [d]	0.875 [d]	0.499 [d]	0.660 [d]	0.600 [d]	0.677 [e]	0.170 [e]

Weights were recorded at the same time each day (morning), except on day 6: evening. [a] Start undoped gels in 0, 100 and 300 mg/kg cohorts; [b] Start treatment with 0, 100 or 300 mg/kg C16 using medicated gels; [c] Animals were fasted during night between days 6 and 7; Statistics: [d] ANOVA, [e] Kruskal-Wallis.

Table W. Daily gel or water consumption during repeat-dose toxicity study.

**Cohort**	**Average consumption** (g/animal) [a]										
	**Day -3**	**Day -2** [b]	**Day -1**	**Day 0** [c]	**Day 1**	**Day 2**	**Day 3**	**Day 4**	**Day 5**	**Day 6**	**Day 7**
0 mg/kg			10.1	10.7	13.5	15.0	11.8	11.7	18.0	12.5	13.4
100 mg/kg			10.4	9.4	14.9	12.3	11.2	15.1	18.1	13.1	11.8
300 mg/kg			8.2	14.7	15.2	12.6	14.7	12.9	15.4	13.6	12.9
Water [d]					7.0	9.8	7.0	7.2	10.0	8.1	9.7

Consumption was recorded at the same time each day (morning). [a] Relative overestimation gel consumption due to removal debris from bedding material and fecal matter. [b] Start undoped gels in 0, 100 and 300 mg/kg cohorts; [c] Start treatment with 0, 100 or 300 mg/kg C16 in medicated gels; [d] Animals were fasted during night between days 6 and 7.

Table X. Feed consumption during repeat-dose toxicity study.

Cohort	Average consumption (g/animal)										
	Day -3	Day -2 [a]	Day -1	Day 0 [b]	Day 1	Day 2	Day 3	Day 4	Day 5	Day 6	Day 7
0 mg/kg					4.3	5.8	6.3	6.1	6.3	6.0	6.7
100 mg/kg					4.8	5.4	6.1	6.9	5.9	5.6	5.6
300 mg/kg					6.0	6.3	8.4	6.6	6.1	7.1	6.2
Water [c]					5.4	6.7	6.1	5.7	6.1	6.1	

Consumption was recorded at the same time each day (morning). [a] Start undoped gels in 0, 100 and 300 mg/kg cohorts; [b] Start treatment with 0, 100 or 300 mg/kg C16 in medicated gels; [c] Animals were fasted during night between days 6 and 7.

Table Y. Organ weights after repeat-dose toxicity study.

Cohort and animals		Organ weights (g) Liver	Kidneys	Spleen
**0 mg/kg**	1-1	1.86	0.47	0.18
	1-2	1.72	0.44	0.13
	1-3	1.79	0.43	0.19
	1-4	1.55	0.45	0.16
	**Average±SD**	**1.73±0.13**	**0.45±0.02**	**0.17±0.03**
**100 mg/kg**	2-1	2.05	0.46	0.24
	2-2	1.42	0.43	0.12
	2-3	1.84	0.44	0.17
	2-4	1.37	0.46	0.11
	**Average±SD**	**1.67±0.33**	**0.45±0.02**	**0.16±0.06**
**300 mg/kg**	3-1	1.63	0.39	0.15
	3-2	1.97	0.41	0.21
	3-3	1.67	0.41	0.13
	3-4	1.52	0.36	0.11
	**Average±SD**	**1.70±0.19**	**0.39±0.02***	**0.15±0.04**
	p	0.905	0.001	0.844

* Statistics: ANOVA with post-hoc Tukey HSD.

Table Z. Clinical chemistry and hematology parameters from the repeat-dose toxicity test.

Parameter	Unit	Normal reference values		Cohort																		
				0 mg/kg					100 mg/kg					300 mg/kg					Water			
		Avg±SD	Range	1-1	1-2	1-3	1-4	Avg±SD	2-1	2-2	2-3	2-4	Avg±SD	3-1	3-2	3-3	3-4	Avg±SD	4-1	4-2	4-3	Avg±SD
**Clinical chemistry**																						
ALP	IU/L	35-96 [a]		74.4	99.0	96.5	97.2	**92±12**	72.0	107.9	131.0	160.3	**118±33**	103.7	72.5	112.8	85.8	**94±18**	84.0	85.7	109.5	**93±14**
ALT	IU/L	17-77 [a]		60.0	16.8	40.7	52.4	**43±19**	45.4	115.3	29.8	91.1	**70±40**	40.6	38.3	55.6	36.2	**43±9**	59.5	55.1	119.0	**78±36**
AST	IU/L	196±133 [b]	59-382	234.4	62.2	222.6	231.2	**188±84**	125.6	884.6	120.7	248.0	**345±365**	189.4	163.0	311.9	220.0	**221±65**	359.8	142.2	894.8	**466±387**
CHO	mg/dL	91±59 [b]	65-188	111.2	84.8	96.2	71.3	**91±17**	75.3	82.9	106.3	120.3	**96±21**	50.4	91.3	84.6	66.8	**73±18**	83.6	99.9	88.1	**91±8**
GLUC	mg/dL	229±34 [c]	169-298	215.2	212.4	207.1	189.8	**206±11** [e]	184.7	256.6	215.6	214.5	**218±30** *	180.8	217.0	233.3	192.8	**206±24** [e]	138.6	191.8	78.6	**136±57**
TP	g/L	44±11 [d]		37.6	38.4	38.8	36.4	**37.8±1.0**	41.9	35.8	46.3	44.0	**42.0±4.5**	0.0	38.5	44.4	43.6	**31.6±21.3**	43.4	49.8	43.8	**45.7±3.6**
TRIGL	mg/dL	104±38 [b]	58-156	189.2	205.0	313.9	122.4	**207±79**	135.1	54.2	154.4	95.1	**110±45**	81.2	88.1	122.6	200.2	**123±55**	131.5	91.0	101.0	**108±21**
**Hematology**																						
WBC	$10^3/\mu L$	8.0±3.2 [b]	3.9-11.9	3.34	2.18	2.63	3.93	**3.02±0.77**	n/a	n/a	3.10	2.99	**3.05±0.08**	n/a	2.3	3.63	n/a	**2.97±0.94**	n/a	1.62	n/a	**1.62**
RBC	$10^6/\mu L$	9.11±0.70 [b]	8.21-10.01	8.87	7.29	6.11	5.78	**7.02±1.40**	n/a	n/a	7.83	8.81	**8.32±0.69**	n/a	4.96	7.67	n/a	**6.32±1.92**	n/a	8.09	n/a	**8.09**
HGB	g/Dl		10.2-16.6 [b]	13.9	11.9	10.1	9.4	**11.3±2.0**	n/a	n/a	12.3	12	**12.2±0.2**	n/a	8.0	12.1	n/a	**10.1±2.9**	n/a	13.0	n/a	**13**
HCT	%	42.6±3.2 [b]	39.1-46.9	46.9	39.4	33.2	31.2	**37.7±7.1**	n/a	n/a	40.8	48.1	**44.5±5.2**	n/a	28.5	39.5	n/a	**34.0±7.78**	n/a	40.9	n/a	**40.9**
MCV	Fl	46.8±1.8 [b]	44.5-49.5	52.9	54.0	54.3	54	**53.8±0.6**	n/a	n/a	52.1	54.6	**53.4±1.8**	n/a	57.5	51.5	n/a	**54.5±4.24**	n/a	50.6	n/a	**50.6**
MCH	pg	17.0±0.8 [b]	16.1-17.9	15.7	16.3	16.5	16.3	**16.2±0.4**	n/a	n/a	15.7	13.6	**14.7±1.5**	n/a	16.1	15.8	n/a	**16.0±0.2**	n/a	16.1	n/a	**16.1**
PLT	$10^3/\mu L$		160-410 [b]	231	304	217	281	**258±41**	n/a	n/a	304	258	**281±33**	n/a	257	274	n/a	**2665±12**	n/a	338	n/a	**338**
NEUT	%	19±9 [b]	10-33	n/a	25.2	31.9	n/a	**28.6±4.7**	n/a	n/a	20.3	13.4	**16.9±4.9**	n/a	n/a	39.9	n/a	**39.9**	n/a	43.2	n/a	**43.2**
LYMP	%	77±11 [b]	63-88	n/a	73.9	67.7	54.6	**65.4±9.9**	n/a	n/a	76.8	81.6	**79.2±3.4**	n/a	n/a	60.1	n/a	**60.1**	n/a	56.8	n/a	**56.8**
MONO	%	2±2 [b]	0-5	3	0.9	0	1.5	**1.4±1.3**	n/a	n/a	2.9	2	**2.5±0.6**	n/a	5.2	0	n/a	**2.6±3.7**	n/a	0	n/a	**0**
EO	%	1±1 [b]	0-2	0.9	0	0.4	n/a	**0.4±0.5**	n/a	n/a	0	0	**0±0**	n/a	0	0	n/a	**0±0**	n/a	0	n/a	**0**
BASO	%	0±0 [b]	0-0	0.3	0	0	0	**0.08±0.2**	n/a	n/a	0	0.3	**0.2±0.2**	n/a	0	0	n/a	**0±0**	n/a	0	n/a	**0**
NRBC	$10^3/\mu L$			0	0	0	0	**0±0**	n/a	n/a	1.5	0	**0.75±1.06**	n/a	0	0.7	n/a	**0.35±0.49**	n/a	1.2	n/a	**1.2**
IG	$10^3/\mu L$			n/a	0	0	n/a	**0±0**	n/a	n/a	0	0.08	**0.04±0.06**	n/a	n/a	0	n/a	**0**	n/a	0	n/a	**0**

Abbreviations: Avg: Average; WBC: White blood cells; RBC: Red blood cells; HGB: Hemoglobin; HCT: Hematocrit; MCV: Mean corpuscular volume; MCH: Mean corpuscular hemoglobin; PLT: Platelets; PCT: Platelet hematocrit; ALP: Alkaline phosphatase; ALT: Alanine transaminase; AST: Aspartate transaminase; CHO: Cholesterol; ; GLUC: Glucose; IG: Immature granulocytes; NRBC: Nucleated red blood cells; TP: Total protein; TRIGL: Triglycerides; [a] Normal reference values according to the University of Minnesota;[2] [b] Normal reference values according to Wolford et al.;[3] [c] Normal reference values according to Serfilippi et al.;[4] [d] Normal reference values according to Fox et al.;[5] [e] Marginally significant difference in glucose level between 0 mg/kg and water cohort (*p*= 0.062), and between 300 mg/kg and water cohort (*p*= 0.063); * Significant difference between 100 mg/kg and water cohort (*p*= 0.028).

E.C. Whole blood C16 levels

The concentration of C16 in the individual hemolyzed mouse blood samples is presented in Table AA and Table BB.

Table AA. Individual blood concentration (ng/mL) for C16 in cohorts on medicated gel.

Cohort and animal		Evening	Morning
**0 mg/kg**	1-1	BLOQ	BLOQ
	1-2	BLOQ	BLOQ
	1-3	0.862	BLOQ
	1-4	1.54	0.672
	**Average±SD**	**1.20±0.479**	**0.672**
**100 mg/kg**	2-1	8.39	50.8
	2-2	5.39	11.5
	2-3	9.33	6.14
	2-4	10.8	56.2
	**Average±SD**	**8.48±2.28**	**31.2±26.0**
**300 mg/kg**	3-1	3.51	30.1
	3-2	NS	21.6
	3-3	13.3	79.3
	3-4	6.09	21.3
	**Average±SD**	**7.63±5.07**	**38.1±27.8**

BLOQ: below the limit of quantitation (0.5 ng/mL). Samples that were below the limit of quantification were not used in the calculation of averages.

Table BB. Individual dosing details and individual and average whole blood concentrations (ng/mL) for C16 after single 300 mg/kg dosing via oral gavage.

	**Animal**			
	**4-1**	**4-2**	**4-3**	**Average±SD**
Animal weight (g)	28.18	31.37	29.26	
Volume dosed (µL)	282	314	293	
Time (h)				
0.5	19.3	60.2	17.5	32.3±24.2
1	28.5	40.3	16.2	28.3±12.1
3	18.4	13.9	13.1	15.1±2.9

F. References

1. Roman BI, De Ryck T, Patronov A, Slavov SH, Vanhoecke BWA, Katritzky AR et al. 4-Fluoro-3',4',5'-trimethoxychalcone as a new anti-invasive agent. From discovery to initial validation in an in vivo metastasis model. Eur J Med Chem. 2015; 101:627-639. PMID: 26204510. DOI: 10.1016/j.ejmech.2015.06.029.

2. Research Animal Resources. Reference Values for Laboratory Animals [Internet]. Unversity of Minnesota, [cited 2017 Jun 7]. Available from: http://www.ahc.umn.edu/rar/refvalues.html/.

3. Wolford ST, Schroer RA, Gohs FX, Gallo PP, Brodeck M, Falk HB et al. Reference range data base for serum chemistry and hematology values in laboratory animals. J Toxicol Environ Health. 1986; 18(2):161-88. PMID: 3712484. DOI: 10.1080/15287398609530859.

4. Serfilippi LM, Pallman DR, Russell B. Serum clinical chemistry and hematology reference values in outbred stocks of albino mice from three commonly used vendors and two inbred strains of albino mice. Contemp Top Lab Anim Sci. 2003; 42(3):46-52. PMID: 19760836.

5. Fox JG, Barthold S, Davisson M, Newcomer CE, Quimby FW, Smith, A, editors. The mouse in biomedical research, 2nd ed. Vol. 3: Normative biology, husbandry, and models. Burlington: Academic Press; 2006. 816 p.
